# Fertilization Alters Indicator Species Serving as Bioindicators for Evaluating Agricultural Practices Related to Maize Grain Yield

**DOI:** 10.3390/microorganisms13061384

**Published:** 2025-06-13

**Authors:** Guoqiang Li, Jiaqing Liu, Wenya Zhang, Jvshui Hu, Peng Shi, Gehong Wei

**Affiliations:** State Key Laboratory for Crop Stress Resistance and High-Efficiency Production, Shaanxi Key Laboratory of Agricultural and Environmental Microbiology, College of Life Sciences, Northwest A&F University, Yangling 712100, China; guoqiangli@nwafu.edu.cn (G.L.); 18740411203@163.com (J.L.); 2023056378@nwafu.edu.cn (W.Z.); hujvshui@163.com (J.H.)

**Keywords:** agricultural practices, soil microbial community, fertilization, indicator species, maize productivity

## Abstract

Diversified agricultural practices reconfigure agroecosystem services by modifying fertilization, tillage intensities, and cropping patterns, altering soil properties and microbial assemblages. However, microbial communities, as critical bioindicators of soil health and productivity, respond to agricultural disturbances, and the effects of multiple practices on productivity-associated indicator species require further validation. Using 16S and ITS amplicon sequencing, this study employed a field experiment to investigate the effects of agricultural practices on soil properties, maize productivity, and microbial communities under two fertilization treatments. Within each treatment, we assessed correlations between indicator species associated with cropping–tillage practices and soil productivity. Results showed that fertilization significantly altered soil properties, increased maize grain yield by 23.9%, and reshaped bacterial and fungal community structures, increasing bacterial richness by 23% but reducing fungal richness and Shannon index by 15% and 20%, respectively. Furthermore, cropping–tillage practices significantly affected microbial communities and grain yields in both fertilized and unfertilized treatments despite a slight influence on soil properties. Distinct sets of bacterial and fungal indicator species were identified for each fertilization treatment: unfertilized soils harbored 21 dominant bacterial indicator species (e.g., *Bacillus*, *Rhizobium*, *Streptomyces*) and 8 fungal indicators (e.g., *Cryptococcus*, *Gibberella*, *Tetracladium*); fertilized soils contained 24 dominant bacterial indicators (e.g., *Fusobacterium*, *Clostridium*, *Lactobacillus*) and 6 fungal indicators (e.g., *Gibberella*, *Cladosporium*, *Mortierella*). Notably, abundances of specific indicator genera (e.g., bacteria: *Bacteroides*, *Gemmatirosa*, *Iamia*, *Lysobacter*, *Prevotella*, *Staphylococcus*, *Sutterella*; fungi: *Glomus*, *Fusicolla* in unfertilized soil; bacteria: *Dinghuibacter*, *Haliangium*, *Kribbella*, *Rhodomicrobium*, *Terrimonas*; fungi: *Pulvinula* in fertilized soil) correlated positively with grain yields. These findings demonstrate that fertilization reshapes the composition of microbial indicator species significantly associated with maize productivity. Tailored microbial indicator assemblages specific to distinct fertilization strategies are therefore essential for evaluating crop productivity and assessing agricultural practice impacts. Consequently, monitoring these indicator species enables rapid assessment of soil fertility changes, offering guidance for fertilization management.

## 1. Introduction

Excessive agricultural practices accelerate changes in ecosystem functions and services [1], with resulting soil nutrient deficiency and biodiversity loss severely constraining intensive farming and agricultural production [2,3]. Agricultural practices face the dual challenge of preserving soil health and increasing productivity [4]. Fertilization, intercropping systems, and film mulching techniques have been widely adopted to enhance local food production, such as in the Loess Plateau [5,6,7]. However, each practice harbors distinct disadvantages: excessive fertilizer dependence accelerates land degradation [8], while traditional tillage without chemical inputs struggles to meet food security demands due to low yields. Thus, reasonable management strategies should be established to balance conflicts between food production and agricultural sustainability. For this purpose, soil physicochemical or biological parameters serve as productivity indicators [4] to guide farmers in identifying “moderate” agricultural practices.

Imbalanced soil nutrients contribute to declines in soil health and productivity [9,10], severely constraining crop growth, reducing yields, and threatening food security [11]. Studies have demonstrated significant correlations between soil health, productivity, and nutrient levels [12]; thus, nutrient cycling determines nutrient availability and supply capacity in agricultural ecosystems [13]. Fertilizers, particularly nitrogen (N), phosphorus (P), and potassium (K), significantly impact soil microbial communities in farmland soil. Increasing nitrogen alters the soil carbon-to-nitrogen ratio, favoring nitrogen-assimilating microbes and significantly increasing *Pseudomonadota* and *Patescibacteria* relative abundance while decreasing Acidobacteria [14]. Phosphorus promotes the enrichment of phosphorus-cycle-related microbial groups and enhances soil phosphorus availability via secretion [15]. Appropriate potassium stabilizes microbial community structure via potassium ions regulating microbial cell osmotic pressure and enzyme activity, leading to a marked increase in Actinobacteria abundance [16]. Moreover, microorganisms are pivotal in the cycling of carbon (C) [17], nitrogen [18,19], phosphorus [20,21], and other soil elements. They directly influence nutrient dynamics and soil productivity by regulating microbial biomass [22,23], activity [24], functional gene diversity [25,26], and specific taxa [27]. These microbial parameters serve as sensitive indicators of soil ecosystem vitality and functionality [28,29], enabling an in-depth assessment of soil health and its responses to environmental changes through monitoring. Furthermore, crop yield, a direct indicator of soil health and productivity, correlates significantly with these parameters [12]. Fertilization dominates agricultural measures for adjusting and optimizing soil nutrient availability [30,31]. It supplements crop nutrients, improving nutrient use efficiency and enhancing crop productivity [32]. Soil nutrient availability (e.g., carbon [33], nitrogen [34], and phosphorus [35]) has been proposed as an indicator of soil productivity, enabling tailored agricultural practices to specific soil quality and yield targets. China’s Soil Testing and Formula Fertilization Project (STFFP) customizes management based on crop needs, soil nutrients, and yield targets to optimize fertilizer efficiency and sustainably enhance agricultural productivity [36]. However, current agricultural strategies predicated on biochemical indicator regulation face challenges: soil nutrients exhibit hysteresis in response to management practices [37], creating mismatches between conventional indicators and modern agriculture’s dynamic demands.

Soil microorganisms constitute key components of biodiversity and play pivotal roles in driving ecological processes within agroecosystems [38]. Particularly, bacterial and fungal communities play crucial roles in agricultural soils [39]. These microbial communities regulate soil nutrient cycles through organic matter decomposition and biogeochemical transformations of essential elements, including nitrogen and phosphorus [40]. The soil microbiome also serves as a vital determinant of plant growth and health by facilitating nutrient uptake [41], mitigating abiotic stress [42], and suppressing phytopathogens [43]. Soil microbial structure and their functions directly influence their participation in soil nutrient cycles, thereby significantly determining agricultural productivity [44,45]. Modern agricultural practices, including fertilization regimes, intercropping systems, and film mulching, significantly modulate microbial community succession by altering soil physicochemical properties and nutrient availability. Studies demonstrate that soil microbial communities are sensitive to agricultural interventions due to some microbial groups’ capacity to rapidly detect environmental perturbations [46]. Therefore, specific microbial taxa are expected to serve as reliable indicators of management practice impacts [46]. In maize fields, the relative abundance of key microbial taxa shifts significantly under different fertilization strategies. Long-term application of mineral fertilizers, particularly nitrogen-rich ones, often enriches Proteobacteria [47]. By contrast, soils under organic management frequently exhibit higher relative abundances of Proteobacteria and Bacteroidetes than those under mineral fertilization alone [48]. Furthermore, in integrated organic–inorganic fertilization systems, the dynamics of Firmicutes and Bacteroidetes communities correlate with soil carbon and nitrogen cycling efficiency [49,50]. These findings highlight how the community structure and functional traits of specific microbial taxa reflect the evolution of soil ecological functions in maize systems under distinct fertilization regimes. Clarifying microbial responses to management practices enables the development of microbial indicators for soil productivity assessment, providing timely insights for precision agriculture, particularly in ecologically vulnerable agroecosystems.

Diversified agricultural practices have been extensively implemented to meet the needs of sustainable agriculture [51,52]. These practices modify tillage or fertilization regimes, altering soil properties and microbial communities [53,54]. However, the effectiveness of microbial communities as reliable bioindicators for evaluating agricultural practices remains insufficiently validated. Different fertilization methods and agricultural practices have diverse impacts on the microbial community [30,55], leading to inconsistent relationships between indicator microorganisms and crop yields [56,57]. Thus, selecting appropriate biological indicators to assess specific agricultural production remains challenging [58]. For example, the Loess Plateau widely adopts the combined practice of “fertilization + maize–soybean intercropping + ridge-furrow tillage” [59,60,61,62]. Fertilization primarily influences soil microbial communities by altering soil physicochemical properties [63,64,65], but it remains unclear whether these influences alter specific taxonomic groups associated with other practices, potentially compromising their effectiveness as soil productivity bioindicators. We hypothesized that fertilization and cropping–tillage practices have distinct effects on soil properties, maize productivity, and microbial communities. Additionally, fertilization significantly reconfigures specific indicator species of cropping–tillage practices related to maize grain yield.

In this study, we established a two-factor field experiment in rain-fed maize cultivation systems, combining fertilizer treatments (unfertilized vs. fertilized) and cropping–tillage practices (conventional tillage with maize monocropping, conventional tillage with maize–soybean intercropping, ridge-furrow tillage with maize monocropping, and ridge-furrow tillage with maize–soybean intercropping) to create different agricultural practices. We investigated (i) the differential impacts of various cropping–tillage practices on soil properties, maize productivity, and microbial communities under two fertilizer treatments and (ii) the correlations between microbial communities/indicator species and maize grain yields in unfertilized and fertilized soils. This study provides foundations for evaluating the applicability of microbial communities as indicators of agricultural productivity across different agricultural practices.

## 2. Materials and Methods

### 2.1. Site Description and Experimental Design

Field experiments were conducted at the Changwu Agro-Ecological Experimental Station in Shaanxi Province, China (35°14′47″ N, 107°41′45″ E), situated on the Loess Plateau. This region has a semi-arid continental monsoon climate with a mean annual air temperature of 10.1 °C and annual precipitation of 570 mm. Rainfed cropping systems predominate in this region, where maize (*Zea mays* L.) and wheat (*Triticum aestivum* L.) are cultivated as primary crops in annual monocultures. The soil, originating from loess deposits, is classified as silty loam (Cumulic Haplustoll) according to the United States Department of Agriculture (USDA) soil taxonomy system with the following properties: pH 8.25, organic matter 16.68 g kg^−1^, total nitrogen 0.97 g kg^−1^, total phosphorus 1.69 g kg^−1^, available nitrogen 50.87 mg kg^−1^, available phosphorus 26.35 mg kg^−1^, and available potassium 372.92 mg kg^−1^.

**Figure 1:**
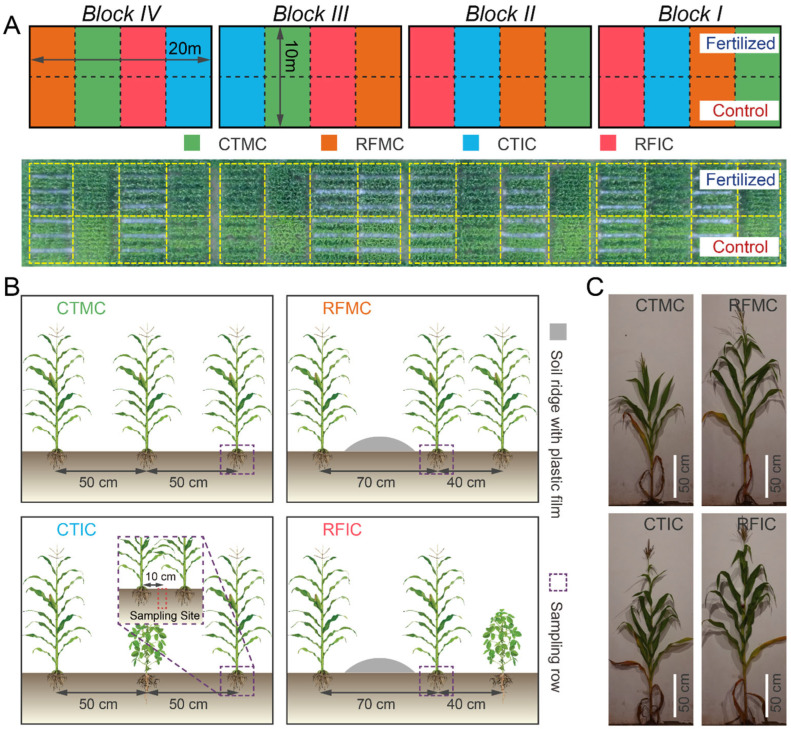
Field layout and treatment details. (**A**) The top panel shows a layout schematic of the four blocks in the experimental field. Plots filled in colors representing four cropping–tillage practices (green: CTMC, blue: CTIC, orange: RFMC, red: RFIC) are arranged in a semi-randomized manner under two fertilizer treatments (control: bottom plots; fertilized: top plots). The bottom panel is an aerial photograph with individual plots outlined in golden dashed lines. (**B**) Details of treatments and sampling sites for cropping–tillage practices. CTMC and CTIC plots contain ten 5 m-long plant rows spaced at 50 cm intervals. For RFMC and RFIC plots, the adjacent row interval is adjusted to 70 cm and 40 cm due to the soil ridges (silver bulges). Purple dashed boxes mark the sampling rows for plant and soil samples within each plot. Red dashed boxes indicate the approximate soil sampling sites. (**C**) Plant phenotypes of cropping–tillage practices in control soils at the tasseling stage.

Field experiments started in 2008 and employed a split-plot design with four replicates, hierarchically structuring two treatment factors: a main-plot factor of fertilizer treatments and a sub-plot factor of cropping–tillage practices. The fertilizer treatments consisted of two levels: no fertilizer (control) and conventional fertilizer application (fertilized). Within each main plot, four cropping–tillage practice treatments were implemented, including conventional tillage with maize monocropping (CTMC), conventional tillage with maize–soybean intercropping (CTIC), ridge-furrow tillage with maize monocropping (RFMC), and ridge-furrow tillage with maize–soybean intercropping (RFIC). These cropping–tillage practices were semi-randomly allocated within each fertilizer main plot. All CTMC plots contained ten maize rows with 50 cm row spacing and 25 cm plant spacing, achieving a density of 170 plants per plot. CTIC plots modified this configuration by replacing three maize rows with soybean rows, resulting in seven maize rows (119 plants) intercropped with three soybean rows (99 plants at 13 cm spacing). The ridge-furrow tillage treatments (RFMC/RFIC) introduced three plastic-mulched ridges (70 cm width), partitioning plots into three 80 cm planting beds. These practices adjusted row spacings to 70 cm and 40 cm while maintaining equivalent plant spacing and density to those in CTMC/CTIC. Therefore, RFMC preserved the planting density of CTMC after spatial rearrangement, while RFIC mirrored the plant distribution of CTIC. Detailed descriptions of cropping–tillage practices and field layouts are illustrated in Figure 1.

### 2.2. Fertilization and Management

Field experiments were conducted over ten consecutive years (2008–2018). To mitigate initial soil heterogeneity, multiple rounds of mechanical land leveling (e.g., deep plowing and rotary tillage) were performed on the plow layer prior to establishing the field experiment to homogenize the soil properties. Analysis of soil pH, organic matter, and nutrients after homogenization indicated no significant differences among experimental blocks (Table S1). This procedure ensured the baseline homogeneity of soil properties and microbial communities. These experimental plots were designed with ridges and furrows according to different treatments and subjected to annual fertilization. Phosphorus (P) and potassium (K) fertilizers were applied as basal dressings, whereas nitrogen (N) fertilizer followed a split application strategy: 40% as basal dressing and 60% as topdressing during the maize trumpet stage. All basal dressings were manually spread evenly on the soil surface and then plowed to incorporate nutrients into the subsurface layer. The fertilizer sources for N, P, and K were urea (46% N), calcium superphosphate (16% P_2_O_5_), and potassium sulfate (51% K_2_O), applied annually at rates of 180 kg N, 120 kg P_2_O_5_, and 100 kg K_2_O per hectare, respectively. Soil ridges mulched with transparent plastic films were additionally installed throughout the entire maize season to maintain the soil moisture and temperature. The plastic films were removed at the harvest stage and remulched before seeding in the following year. Crop residues were also removed at the annual harvest stage. In the first week of May, maize (Zhengdan 958) and intercropped soybean (Zhonghuang 13) were sown using a hand-powered hole-seeding machine. Weed control measures were manually performed three or four times by local farmers. All other agronomic practices were standard and uniform across all plots. Natural rainfall served as the sole water source for maize growth. Meteorological parameters during the maize growing season of the sampling years are presented in Figure S1.

### 2.3. Sample Collection and Processing

In this study, plant and soil samples were collected to determine maize productivity, soil properties, and microbial community. To minimize experimental and sampling variability, we implemented a standardized row configuration system where maize rows in CTMC/RFMC and CTIC/RFIC plots sharing equivalent spatial positions were designated as core experimental rows. The soybean rows in CTIC/RFIC plots and their spatially corresponding maize rows in CTMC/RFMC plots served, respectively, as supplementary experimental rows (see Figure 1 for spatial details). This standardized spatial arrangement effectively balanced the confounding effects of the differences in cropping–tillage practices while maintaining consistent micro-environmental conditions for plant and soil assessments. Furthermore, all sampling and measurements were strictly confined to these predefined experimental rows, which were established as standardized sampling rows. This approach ensured methodological consistency in quantifying plant and soil parameters across different agricultural practices.

In the maturing stage of 2017 and 2018, two maize plants were randomly selected from the sampling rows of each experimental plot for height and biomass measurement. Plant height was measured using a measuring scale. After removing the roots, the plant samples were chopped and oven-dried at 85 °C to constant weight for straw biomass determination. For grain yield assessment, ten consecutive maize plants were randomly selected from one of the sampling rows in each plot to evaluate grain yields at physiological maturity. Ears were detached from stalks, and the grains were manually threshed. These threshed grains were then dried at 105 °C, and the grain yields were calculated at 14% moisture content, following USDA standards.

Soil samples were collected across all experimental plots during the maturing stage of 2018. From each plot, three standardized sampling rows were selected for soil collection. Within these rows, six representative soil cores (5 cm diameter and 20 cm depth) were systematically obtained using a steel auger at random positions within plant spacings, maintaining a distance of about 10 cm from maize stems to minimize rhizosphere disturbance. The collected cores from each plot were homogenized to form a composite sample (Figure 1B). Composite samples were divided into two subsamples and stored in individual polyethylene bags. These subsamples were placed in a cooler and immediately transported to the laboratory. Subsamples were then stored at −80 °C and at −20 °C for DNA extraction and characterizing soil physicochemical properties, respectively. We selected some soil properties, mainly focused on fertility parameters critical for soil assessment. Standard soil testing procedures and previous reports were followed to quantify the soil moisture content (105 °C drying), pH (1:2.5 soil:water suspension), total carbon/nitrogen (TC/TN, Elementar vario MAX cube CNS analyzer, Elementar, Langenselbold, Germany), dissolved organic carbon (DOC, 0.5 mol L^−1^ K_2_SO_4_ extraction-Elementar vario TOC cube TOC analyzer, Elementar, Langenselbold, Germany), ammonium/nitrate-nitrogen (NH_4_^+^-N/NO_3_^−^-N, 2 mol L^−1^ KCl extraction-SEAL AA3 HR AutoAnalyzer, SEAL Analytical GmbH, Norderstedt, Germany), and microbial biomass carbon/nitrogen (MBC/MBN, chloroform fumigation-extraction method-Elementar vario TOC cube TOC analyzer, Elementar, Langenselbold, Germany).

### 2.4. DNA Extraction, PCR, and High-Throughput Sequencing

Soil DNA extraction, DNA concentration and purity determination, and specific gene amplification were performed for library preparation and amplicon sequencing. Total genomic DNA was extracted from 0.5 g soil samples using the FastDNA^®^ SPIN Kit for Soil (MP Biochemicals, Solon, OH, USA) following the manufacturer’s protocols. Each sample was extracted twice and pooled to yield enough DNA. DNA concentration and purity were assessed using the NanoDrop 2000 (Thermo Scientific, Waltham, MA, USA) and 1% (*w*/*v*) agarose gel electrophoresis (Bio-Rad, Hercules, CA, USA). The V3-V4 region of the bacterial 16S rRNA gene and the fungal internal transcribed spacer 1 (ITS1) gene were applied for polymerase chain reaction (PCR) amplification using the primer pairs 338F (5′-ACTCCTACGGGAGGCAGCAG-3’)/806R (5’-GGACTACHVGGGTWTCTAAT-3’) and ITS5-1737F (5’-GGAAGTAAAAGTCGTAACAAGG-3’)/ITS2-2043R (5’-GCTGCGTTCTTCATCGATGC-3’), respectively [66,67]. PCR products were purified using the QIAquick PCR Purification Kit (QIAGEN, Hilden, Germany) after being inspected on 2% agarose gel for the correct size and appropriate concentration. The purified amplicons were pooled into libraries at equimolar concentrations, and the libraries were sequenced on an Illumina platform (Illumina Inc., San Diego, CA, USA). PCR amplification, library preparation, and high-throughput sequencing were performed according to the standard protocols provided by a biotechnology company (Novogene, Shanghai, China).

### 2.5. Bioinformatics Analysis

Raw sequence data were analyzed using bioinformatics approaches to generate amplicon sequence variants (ASVs), followed by taxonomic classification and filtration of low-abundance ASVs. The raw datasets of bacterial and fungal sequences were processed using the divisive amplicon denoising algorithm 2 (DADA2) bioinformatics pipeline to ensure analytical reusability and reproducibility [68]. Based on the DADA2 workflows (version 1.14.1), low-quality sequences were truncated, and the sequence length was trimmed. Forward and reverse sequences with more than three expected errors were discarded. After sequence denoising, pair merging, and chimera detection, ASVs were determined from raw sequence datasets. The ASV taxonomy classification was performed using the RDP Classifier (version 2.11) [69] with an 80% confidence threshold, based on the Silva database (version 128, https://www.arb-silva.de, accessed on 28 July 2021) for bacterial assignment [70] and the UNITE database (version 7.2, https://unite.ut.ee, accessed on 28 July 2021) for fungal assignment [71]. Based on taxonomic assignments, bacterial ASVs classified as Archaea, mitochondria, and chloroplasts were removed. Fungal ASVs unassigned to Kingdom Fungi were also removed. To minimize sequencing errors and low-prevalence features for downstream analysis, bacterial ASVs with less than five counts and fungal ASVs with less than two counts were discarded in at least four samples (number of replicates per treatment). The resulting bacterial and fungal count tables were normalized to the sample with the lowest number of sequences, and subsequent analyses were based on these ASV count tables.

### 2.6. Statistical Analysis

All data analyses were conducted in R (version 3.6.3) unless otherwise specified. Statistical significance was determined at a significance level of *p* < 0.05. Linear mixed-effects models (LMMs) were employed to evaluate the responses of cropping–tillage practices related to soil properties, maize productivity, major phyla, and alpha diversity under control and fertilized treatments. In these models, cropping–tillage practices were set as fixed effects, while block was treated as a random effect [72]. The linear mixed model formula was response variables ~ cropping–tillage practice + (1|block). Statistical significance was assessed using a type II analysis of variance (ANOVA) with Kenward–Roger approximation of the degrees of freedom based on the LMMs. The marginal R^2^ (fixed effects only) and conditional R^2^ (both fixed and random effects) were calculated for each LMM.

Alpha diversity indices (ASV richness and Shannon index) were calculated across rarefaction depths [73]. For beta diversity analyses, ASVs were normalized via the trimmed mean of M values (TMM) method [74] and expressed as counts per million (CPM). Bacterial and fungal community dissimilarities were quantified using Bray–Curtis distances. Unconstrained principal coordinate analyses (PCoA) were conducted to quantify major variance components [75]. Treatment effects (fertilizer treatments and cropping–tillage practices) on the Bray–Curtis dissimilarities of bacterial and fungal communities were evaluated using permutational multivariate ANOVA (PERMANOVA) and analysis of similarities (ANOSIM) with 10^4^ permutations [76]. Subsequently, constrained analysis of principal coordinates (CAP) visualized cropping–tillage practice effects within each fertilizer subgroup for bacterial and fungal communities [76]. The effects of different cropping–tillage practices on Bray–Curtis dissimilarity were further assessed using PERMANOVA and ANOSIM [76].

Distance–decay relationships were quantified using linear least-squares regression between pairwise maize productivity differences (ΔProductivity) and microbial community similarity (1 − Bray–Curtis dissimilarity). Bacterial and fungal ASVs with relative abundances ≥ 0.1% in more than 75% of cropping–tillage practice samples were identified as dominant taxa (persistent species). To identify the ASVs associated with cropping–tillage practices, indicator species analyses were performed to calculate the point-biserial correlation coefficient of an ASV’s positive association with one or a combination of cropping–tillage practices with 10^4^ permutations under two fertilizer treatments. Bipartite networks were constructed using the Fruchterman–Reingold layout and were employed to visualize the significant ASVs (*p* < 0.05) obtained from the indicator species analyses [77]. Indicator species were classified into dominant and sensitive profiles. Indicator species defined as dominant taxa are classified as dominant indicator species, whereas the remaining ones are categorized as sensitive indicator species. Spearman’s correlation coefficients of the bacterial and fungal indicator species and maize grain yield were calculated at the ASV level. The strong (both positive and negative, |*r*| > 0.5) and significant (*p* < 0.05) indicator species correlated with grain yields under different fertilizer treatments were identified. The total relative abundance of each strong and significant indicator species was summed, and the relationships between the total relative abundance and maize grain yield were identified via linear regression.

## 3. Results

### 3.1. Fertilization and Cropping–Tillage Practice Effects on Soil Properties and Maize Productivity

The linear mixed-effects model analysis based on all samples showed that fertilization had a strong effect on soil properties, including pH, carbon-to-nitrogen ratio (C:N), DOC, NH_4_^+^-N, NO_3_^−^-N, MBC, and MBN, whereas cropping–tillage practices had a minimal impact, significantly affecting NH_4_^+^-N only (Table S2). Specifically, soil pH, C:N, and DOC significantly decreased, while NH_4_^+^-N, NO_3_^−^-N, MBC, and MBN significantly increased in fertilized soils (Figure S2). In addition, the results indicated that only the content of NH_4_^+^-N exhibited a significant difference in control soils, whereas no significant variations were observed in the fertilized soils (Table 1).

**Figure 2:**
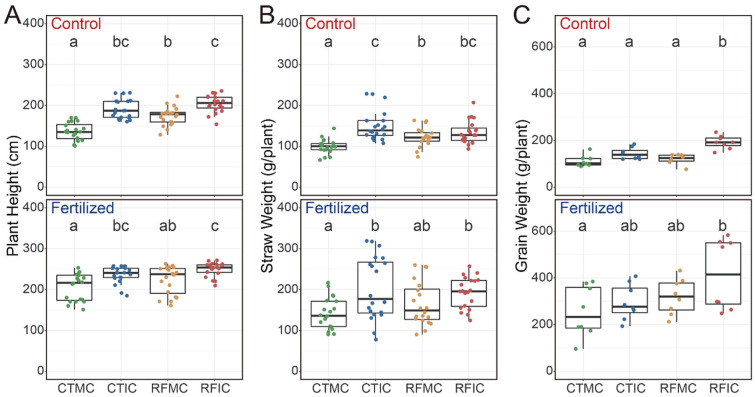
Variations in (**A**) plant height, (**B**) straw weight, and (**C**) grain weight across different cropping–tillage practices based on linear mixed-effects models under control and fertilized treatments. The linear mixed-effects model formula was maize productivity ~ cropping–tillage practice + (1|block). Statistical significance was assessed using type II ANOVA with Kenward–Roger approximation of the degrees of freedom. Boxplots that do not share a letter are significantly different.

Our results indicated that both fertilization and cropping–tillage practices had significant effects on plant height, straw weight, and grain weight (Table S3). Fertilization significantly enhanced plant height, straw weight, and grain weight (Figure S3; Table S3), with average values of 228.75 cm, 152.63 g plant^−1^, and 175.74 g plant^−1^, respectively, in the fertilized soils, in contrast to 176.50 cm, 126.71 g plant^−1^, and 141.78 g plant^−1^ in the control soils. Compared to the control soils, plant height across cropping–tillage practices in fertilized soils showed substantial increases of 61.00 cm (CTMC), 36.96 cm (CTIC), 42.25 cm (RCMC), and 41.04 cm (RFIC; Figure 2A). The straw weight in fertilized soils increased by 37.79 g plant^−1^ (CTMC), 43.38 g plant^−1^ (CTIC), 39.92 g plant^−1^ (RFMC), and 52.06 g plant^−1^ (RFIC), respectively (Figure 2B). Additionally, grain yields under fertilized treatments showed increases of 143.75 g plant^−1^ (CTMC), 152.63 g plant^−1^ (CTIC), 200.00 g plant^−1^ (RFMC), and 223.13 g plant^−1^ (RFIC) when compared to the control soils (Figure 2C). Furthermore, cropping–tillage practices also exerted significant influences on maize productivity parameters under each fertilizer treatment. In both control and fertilized soils, the RFIC plots yielded the highest values, whereas the CTMC yielded the lowest values in terms of plant height, straw weight, and grain weight, with significant differences observed (Figure 2).

### 3.2. Microbial Community Across Cropping–Tillage Practices in Soils

We conducted bacterial and fungal community profiling of soil samples to investigate soil microbial communities. After discarding low-abundance ASVs, we obtained 2417 bacterial and 790 fungal ASVs across all samples for subsequent analysis. Bacterial and fungal communities were analyzed based on normalized ASV count tables, which were rarefied to 23,675 and 57,219 counts, respectively, to standardize sampling efforts (Figure S4). Overall, Proteobacteria (567 ASVs) represented the most abundant bacterial phylum, comprising 23.46% of all bacterial ASVs, followed by Acidobacteria (509 ASVs), Actinobacteria (325 ASVs), Chloroflexi (282 ASVs), Bacteroidetes (210 ASVs), Gemmatimonadetes (135 ASVs), and Firmicutes (115 ASVs; Figure S5). In addition, the three most abundant fungal phyla were Ascomycota (334 ASVs), Basidiomycota (101 ASVs), and Mortierellomycota (42 ASVs), comprising 42.28%, 12.78%, and 5.32% of all fungal ASVs, respectively (Figure S5).

**Figure 3:**
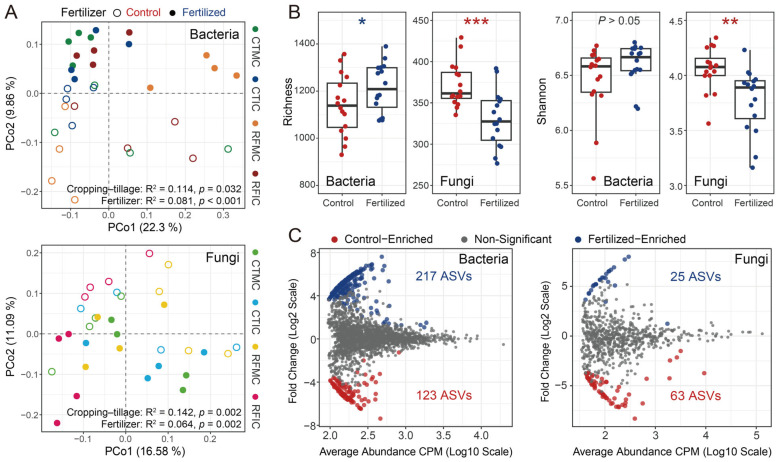
General patterns of microbial communities between control and fertilized soils. (**A**) PCoA showed the bacterial and fungal community structures based on Bray–Curtis dissimilarities. Similarity values for fertilizer and cropping–tillage practices were estimated using PERMANOVA and shown at the bottom of the plots. Shapes indicate different fertilizer treatment samples, while colors represent four cropping–tillage practices. (**B**) Variations in alpha diversity (richness and Shannon index) between different fertilizer treatments. The significance of these differences was estimated using Wilcoxon rank-sum tests. ***: *p* < 0.001; **: *p* < 0.01; *: *p* < 0.05. (**C**) Enrichment of bacterial and fungal communities in control and fertilized soils was determined by differential abundance analysis. Each point represents an individual ASV, and significantly different ASVs are indicated by color points.

Across all samples, the microbial communities in control and fertilized soils differed markedly in bacterial and fungal community structures, alpha diversity, and specific microbial taxa. Unconstrained PCoA based on separate Bray–Curtis dissimilarities of bacterial and fungal communities revealed that soil samples formed distinct clusters in the ordination space, showing clear separations between control and fertilized soils. Bacterial and fungal communities clearly separated along the second principal coordinate axis (PCo2), explaining 9.86% and 11.09% of the total variations attributed to fertilizer application, respectively (Figure 3A). PERMANOVA confirmed these significant differences between fertilizer treatments (bacteria R^2^ = 0.081, *p <* 0.001; fungi R^2^ = 0.064, *p* = 0.002; Table S4). Additionally, ANOSIM verified that bacterial and fungal communities in fertilized soils significantly differed from those in control soils (bacteria: R = 0.168, *p* = 0.002; fungi: R = 0.112, *p* = 0.007; Table S4). For alpha diversity analyses, fertilizer application resulted in a significant increase in bacterial ASV richness, whereas the bacterial Shannon index showed a marginal increase, though not significant (Figure 3B). In contrast, both fungal ASV richness and the Shannon index decreased significantly under fertilizer application (Figure 3B). Furthermore, control and fertilized soils exhibited different microbial enrichment patterns, characterized by specific microbial taxa, based on differential abundance analysis. A total of 340 bacterial ASVs were significantly different, with 123 ASVs enriched in control soils and 217 ASVs enriched in fertilized soils (Figure 3C). These control-enriched ASVs were dominated by Acidobacteria (30.89%, 38 ASVs), Proteobacteria (23.58%, 29 ASVs), and Actinobacteria (11.38%, 14 ASVs), whereas fertilized-enriched ASVs were primarily composed of Proteobacteria (30.89%, 67 ASVs), Acidobacteria (20.28%, 44 ASVs), and Gemmatimonadetes (13.36%, 29 ASVs). For fungal taxonomic profiles, 63 and 25 fungal ASVs were enriched in control and fertilized soils, respectively. Ascomycota constituted the most abundant phylum, accounting for 25.4% (16 ASVs) of the total differentially abundant ASVs in control soils and 20% (5 ASVs) in fertilized soils (Figure 3C). To conclude, the control and fertilized soils exhibited distinct bacterial and fungal community structures, alpha diversity indices, and specific taxa.

Unconstrained PCoA also visualized the differences across different cropping–tillage practices. Subtle clusters were primarily distributed along the first principal coordinate axis (PCo1), accounting for 22.30% and 16.58% of the total variations attributed to cropping–tillage practices for bacterial and fungal communities, respectively (Figure 3A). Both PERMANOVA and ANOSIM analyses confirmed the significant differences among cropping–tillage practices (PERMANOVA: bacteria R^2^ = 0.114, *p* < 0.05; fungi R^2^ = 0.142, *p* = 0.002; ANOSIM: bacteria R = 0.119, *p* = 0.009; fungi R = 0.120, *p* = 0.009; Table S4).

**Figure 4:**
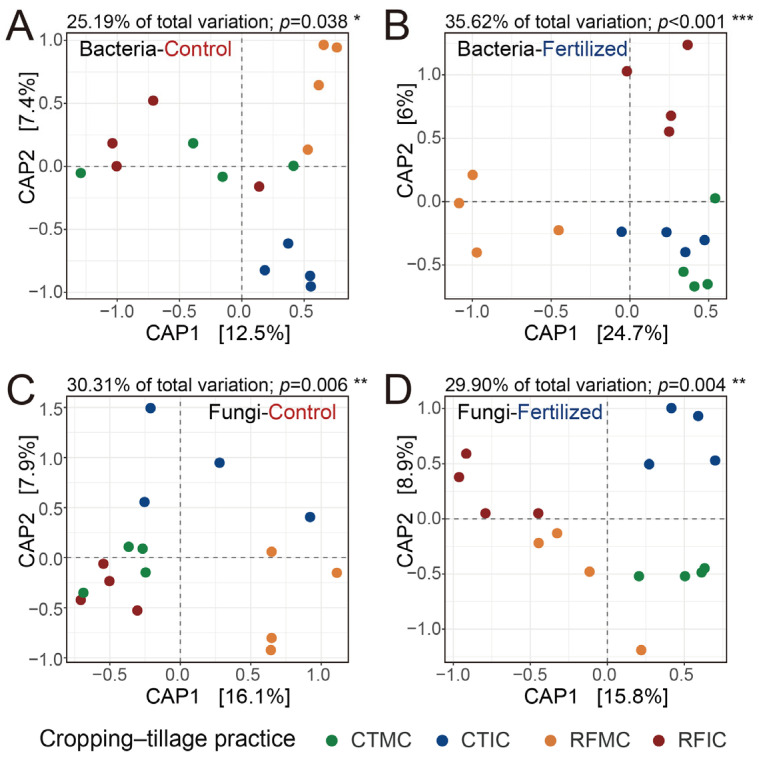
Effects of different cropping–tillage practices on bacterial and fungal communities. Separate CAP ordinations were performed to assess the Bray–Curtis dissimilarities of bacterial communities in control (**A**) and fertilized (**B**) soils, as well as those of fungal communities in control (**C**) and fertilized (**D**) soils. The CAP analyses were constrained by the “cropping–tillage practices” factor. The explained fraction of the total variance is indicated above the plots. Significance was assessed with 10^4^ permutations. ***: *p* < 0.001; **: *p* < 0.01; *: *p* < 0.05. The percentage of variation shown on each axis refers to the explained fraction of total variation. Colored points represent four cropping–tillage practices.

An in-depth analysis of cropping–tillage practice effects on soil microbial communities was performed by assessing the structures of soil bacterial and fungal communities along with alpha diversity. Constrained by the “cropping–tillage practices” factor, separate CAP analyses were performed to investigate the microbial community differences among cropping–tillage practices in both control and fertilized soils. These CAP ordinations visualized distinct clustering and separation in the ordination space and emphasized the effects of cropping–tillage practices on soil bacterial and fungal communities (Figure 4). For bacterial communities, cropping–tillage practices explained 25.19% and 35.62% of the total variation in control and fertilized soils, respectively (Figure 4A,B), whereas for fungal communities, the practices explained 30.31% and 29.90% of the total variation in control and fertilized soils, respectively (Figure 4C,D). Significant differences were confirmed through PERMANOVA analysis in control soils (bacteria: R^2^ = 0.252, *p* < 0.05; fungi: R^2^ = 0.303, *p* = 0.006) and fertilized soils (bacteria: R^2^ = 0.356, *p* < 0.001; fungi: R^2^ = 0.299, *p* = 0.004; Table S5). ANOSIM further validated these significant differences separately in control soils (bacteria: R = 0.203, *p* < 0.05; fungi: R = 0.291, *p* = 0.004) and fertilized soils (bacteria: R = 0.388, *p* = 0.001; fungi: R = 0.307, *p* = 0.003; Table S5). Furthermore, in control soils, no significant differences were observed in bacterial and fungal alpha diversity (ASV richness and Shannon index) across cropping–tillage practices. However, in fertilized soils, cropping–tillage practices showed significant differences in either fungal ASV richness or fungal Shannon index. For bacterial alpha diversity in fertilized soils, the Shannon index differed significantly across cropping–tillage practices, whereas ASV richness showed no significant variations (Table 2). Overall, microbial communities exhibited significant differences across cropping–tillage practices in both control and fertilized soils, with distinct variations observed in their community structures.

### 3.3. Dominant Taxa and Indicator Species Across Cropping–Tillage Practices

The responses of microbial taxa to cropping–tillage practices were assessed in control and fertilized soils. Proteobacteria and Ascomycota were subdivided into their respective classes. For dominant phyla in fertilized soils, the relative abundance of dominant bacterial phyla (>1%) differed significantly across cropping–tillage practices, particularly within Alphaproteobacteria, Deltaproteobacteria, Chloroflexi, Firmicutes, Bacteroidetes, Gemmatimonadetes, and Rokubacteria. In contrast, no significant variation was observed for dominant bacterial phyla in control soils (Figure S6). Regarding fungal phyla, Sordariomycetes and Dothideomycetes varied significantly across cropping–tillage practices in both control and fertilized soils. Distinct responses were also observed in Basidiomycota from control soils, whereas Mortierellomycota differed in fertilized soils (Figure S7). Additionally, to further characterize the responses at the species level, we identified persistent bacterial and fungal species in control and fertilized soils. These species were highly abundant (relative abundance ≥ 0.1%) across 75% of cropping–tillage practice samples and persisted as dominant taxa. A total of 199 dominant bacterial taxa were identified in both the control and fertilized soils, respectively. Among these, control and fertilized soils shared 162 taxa, of which *Sphingomonas*, *RB41*, *Blastococcus*, *MND1,* and *Microvirga* showed greater abundance (Figure S8A). For fungi, 94 dominant taxa were identified in control soils and 81 taxa in fertilized soils, sharing 72 taxa, primarily including *Mortierella*, *Fusarium*, and *Cryptococcus* (Figure S8B).

**Figure 5:**
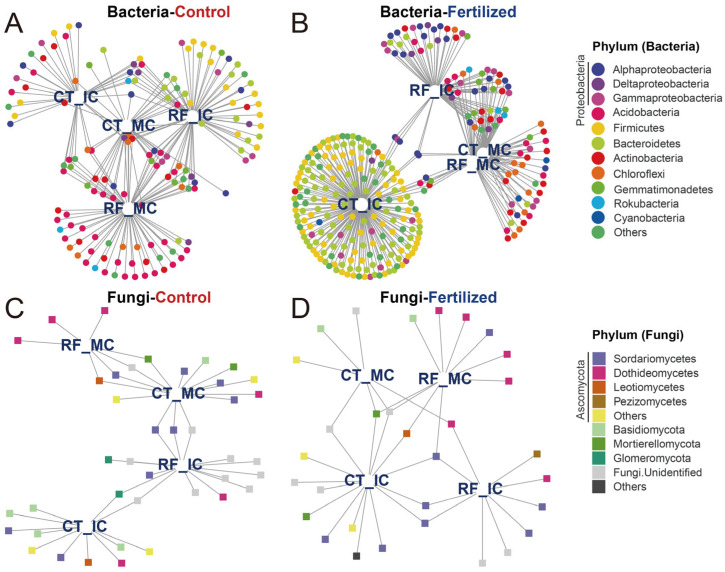
Bipartite networks display cropping–tillage practice-specific ASVs of bacterial and fungal communities in control and fertilized soils as determined using indicator species analysis. Bipartite networks of bacterial indicator species in control (**A**) and fertilized (**B**) soils, and those of fungal indicator species in control (**C**) and fertilized (**D**) soils are shown. Circles represent individual bacteria, and squares represent individual fungi. ASVs that are positively and significantly associated (*p* < 0.05) with one or more of the cropping–tillage practices are connected by lines. ASVs are colored according to their phylum assignment.

Indicator species were identified as being associated with cropping–tillage practices and summarized using bipartite networks. We identified 145 bacterial indicator species in control soils and 293 bacterial indicator species in fertilized soils. These indicator species associated with cropping–tillage practices in control soils primarily belonged to Acidobacteria (38 ASVs, 26.21%), Proteobacteria (25 ASVs, 17.24%), and Firmicutes (19 ASVs, 13.10%; Figure 5A). In fertilized soils, the main indicator species were Bacteroidetes (82 ASVs, 27.99%), Firmicutes (66 ASVs, 22.53%), and Proteobacteria (49 ASVs, 16.72%; Figure 5B). Then, a total of 40 and 31 fungal indicator species were identified in control and fertilized soils, respectively. In control soils, the main fungal indicator species belonged to Ascomycota (21 ASVs, 50.50%) and Basidiomycota (5 ASVs, 12.50%; Figure 5C). The main fungal indicator species associated with cropping–tillage practices in fertilized soils were Ascomycota (19 ASVs, 61.29%) and Basidiomycota (2 ASVs, 6.45%; Figure 5D). These bipartite networks revealed that the patterns were reminiscent of the effects observed in previous microbial communities across cropping–tillage practices. For instance, the high number of bacterial indicator species shared across cropping–tillage practices in control soils reflects their close clustering in ordination analyses. Additionally, the abundance of indicator species specific to RFMC in fertilized soils aligns with its distinct separation in ordination space.

Furthermore, we classified indicator species into dominant and sensitive profiles. Indicator species defined as dominant taxa are classified as dominant indicator species, whereas the others are defined as sensitive indicator species. For bacterial indicator species, 21 dominant indicator species were identified in control soils, including *Fusobacterium*, *Bacillus*, *RB41*, *Microvirga*, *Sphingomonas*, *MND1*, *Rhizobium*, *Chryseolinea*, and *Streptomyces* at the genus level (Figure S9A; Table S6). A total of 24 dominant indicator species were identified in fertilized soils, predominantly *Escherichia*, *Terrisporobacter*, *Fusobacterium*, *Nordella*, *Microvirga*, *MND1*, *dgA-11*, *Prevotellaceae*, *Sphingomonas*, *Clostridium*, *Lactobacillus*, and *Rikenellaceae_RC9* (Figure S9B; Table S6). For fungal indicator species, eight species were identified as dominant indicator species in control soils (Figure S9C; Table S7). Taxa being identified at the genus level included *Cryptococcus*, *Gibberella*, *Humicola*, *Mortierella*, and *Tetracladium*. Six dominant indicator species were obtained in fertilized soils, including *Gibberella*, *Cladosporium*, and *Mortierella* (Figure S9D; Table S8). These dominant indicator species in control and fertilized soils primarily participate in soil organic matter degradation, material cycling, and ecological balance maintenance. Additionally, a total of 104 and 239 bacterial sensitive indicator species were identified in control and fertilized soils, respectively, with 25 overlapping species (Figure S10A), including *Sphaerochaeta*, *Aerococcus*, *Campylobacter*, *Bacteroides*, *Phenylobacterium*, *Lachnospiraceae*, *Sutterella*, *Ruminiclostridium*, *Erysipelotrichaceae*, and *Staphylococcus*. In contrast, 26 and 19 fungal sensitive indicator species were identified in control and fertilized soils, respectively, with no overlapping species (Figure S10B).

### 3.4. Correlations Between Key Taxa and Maize Productivity

Consistent decay in community dissimilarities with partial maize productivity distances was observed for both bacterial and fungal communities (Figure S11). Significant distance–decay relationships were identified between grain yield variation and both bacterial and fungal community dissimilarities (bacterial Bray–Curtis dissimilarity range: 0.29–0.63; fungal Bray–Curtis dissimilarity range: 0.22–0.59). These Bray–Curtis dissimilarity ranges suggest substantial compositional shifts in bacterial and fungal communities. Notably, marginally steeper regression slopes were observed for fungal communities (slope = −0.010, *p* = 0.006) compared to bacterial communities (slope = −0.009, *p* = 0.021; Figure S11C). However, no significant relationships were observed between straw weight variation and bacterial and fungal dissimilarities (Figure S11B). Furthermore, significant distance–decay relationships between the variation in maize height and dissimilarities (slope = −0.06, *p* < 0.001) were only observed for the bacterial community (Figure S11A). Collectively, these findings indicate a significant correlation between microbial community dissimilarities and grain yield variation.

**Figure 6:**
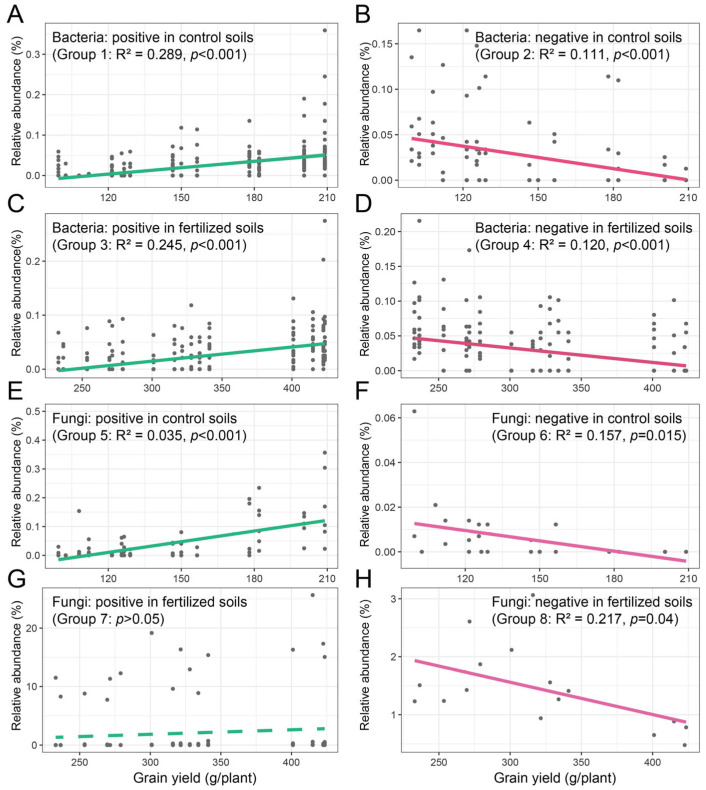
The relationships between bacterial (**A**–**D**) and fungal (**E**–**H**) indicator species and maize grain yield. Linear regression was performed on the relative abundance of the bacterial and fungal indicator species and maize grain yields.

Spearman’s correlation coefficients of the bacterial and fungal indicator species and maize grain yield were calculated at the ASV level. For bacterial indicator species, maize grain yield was positively correlated with *Dongia*, *Lachnospiraceae_NK4A136*, *Bacteroides*, *Prevotella_7*, *Sutterella*, *Ruminiclostridium_6*, *Erysipelotrichaceae_UCG-004*, *Ruminococcus_1*, *Lysobacter*, *Staphylococcus*, *Gemmatirosa*, *Aerococcus*, *Iamia,* and *Allobaculum* (Group 1) and negatively correlated with *Candidatus_Alysiosphaera* (Group 2) in control soils (Table S8). The relative abundance of all positive and negative indicator species was summed, and linear regression was performed on the relative abundance and maize grain yield (Figure 6). The results showed that maize grain yield increased with the abundance of positive indicator species (R^2^ = 0.289, *p* < 0.001) and decreased with the abundance of negative indicator species (R^2^ = 0.111, *p* < 0.001) (Figure 6A,B). Meanwhile, in fertilized soils, maize grain yield was positively correlated with *Kribbella*, *Terrimonas*, *Rhodomicrobium*, *MND1*, *Dongia*, *Dinghuibacter,* and *Haliangium* (Group 3) and negatively correlated with *Nocardioides*, *MND1*, *Nostoc_PCC*, and *Gemmatimonas* (Group 4; Table S8). The results of linear regression showed that maize grain yield increased with the abundance of positive indicator species (R^2^ = 0.245, *p* < 0.001; Figure 6C) and decreased with the abundance of negative indicator species (R^2^ = 0.120, *p* < 0.001; Figure 6D).

Similarly, for fungal indicator species, maize grain yield was positively correlated with *Glomus* and *Fusicolla* (Group 5) and negatively correlated with unidentified Ascomycota (Group 6) in control soils (Table S9). The linear regression was performed on the relative abundance of all positive and negative indicator species and maize grain yield. The results showed that maize grain yield increased with the abundance of positive indicator species (R^2^ = 0.035, *p* < 0.001; Figure 6E) and decreased with the abundance of negative indicator species (R^2^ = 0.157, *p* = 0.015; Figure 6F). Meanwhile, in fertilized soils, maize grain yield was positively correlated with *Pulvinula* (Group 7) and negatively correlated with *Mortierella* (Group 8; Table S9). The results of linear regression did not indicate that maize grain yield increased with the abundance of positive indicator species (*p* > 0.05; Figure 6G) and decreased with the abundance of negative indicator species (R^2^ = 0.217, *p* = 0.04; Figure 6H).

## 4. Discussion

### 4.1. Agricultural Practices Are the Main Drivers of Maize Productivity Improvement and Soil Microbial Community Alteration

Our findings indicated that fertilizer application significantly altered soil properties, including soil pH, C:N, DOC, NH_4_^+^-N, NO_3_^−^-N, MBC, and MBN (Figure S2), while also markedly increasing maize productivity (plant height, straw weight, and grain weight; Figure S3). Regarding microbial communities, fertilizer treatments significantly altered the structures of soil bacterial and fungal community structures (Figure 3A), increased bacterial alpha diversity, decreased fungal alpha diversity (Figure 3B), and altered the enrichment patterns of bacteria and fungi in soils (Figure 3C). Further compositional analysis of the bacterial and fungal taxa significantly enriched in the control and fertilized soils (Figure 3C) revealed that fertilization significantly enriched nitrogen cycle-related bacterial taxa in the soil, including *Nitrosospira*, *Nitrolancea*, and *Rhizobium*, which promote nitrogen transformation and availability [78]. Simultaneously, fertilization-enriched functional taxa involved in complex organic matter degradation, such as *Steroidobacter*, *Lysobacter*, *Bacteroides*, *Pseudomonas*, and *Bacillus*, enhance the decomposition of organic nutrients and elevate MBC and MBN [79]. In addition, nitrogen-fertilized soil specifically enriched microbial taxa involved in phosphorus metabolism, such as *Sphingomonas*, *Flavisolibacter*, and *Gemmatimonas* (key taxa in phosphorus mobilization via organic phosphorus decomposition), indicating a synergistic effect between nitrogen and phosphorus cycling communities [27,80]. These microbial structural shifts directly contributed to the observed changes in soil nutrient status, as evidenced by the increased NH_4_^+^-N, NO_3_^−^-N, MBC, and MBN. Consistent with numerous studies, fertilization influences soil properties and crop growth by regulating the availability of soil mineral nutrients or modifying soil nutrient structure, while soil microbial communities correlate with soil properties and agricultural practices [81].

In contrast, cropping–tillage practices did not significantly alter soil properties (Table 1). However, these practices significantly affected the maize productivity (Figure 2), altered the dominant bacterial and fungal taxa (Figures S7 and S8), and reshaped soil bacterial and fungal community structures (Figure 3A; Table S4). Similar results have also been reported in previous studies [82]. Some researchers speculate that practices like mulching [83] and intercropping [84] fail to significantly enhance the utilization efficiency of soil carbon and nitrogen nutrients or water, which are key factors in altering soil properties. Others have demonstrated that agricultural practices significantly affect microbial community composition [85] and specific microbial taxa [46,85]. A possible explanation is that variations in soil properties induced by cropping–tillage practices were relatively subtle, resulting in insignificant changes. In comparison to soil properties, microbial communities are more sensitive and respond more quickly to changes caused by cropping–tillage practices, leading to significant changes in microbial communities.

### 4.2. Soil Microbial Taxa Reactions to Agricultural Practices Alter Under Different Fertilizer Treatments

Our findings further demonstrate that cropping–tillage practices significantly influenced maize productivity under both control and fertilized conditions, resulting in significant variations across cropping–tillage practices (Figure 2). Concurrently, soil microbial communities in control and fertilized soils were substantially influenced by cropping–tillage practices, exhibiting significant differences in bacterial and fungal community structures (Figure 3). Notably, the impact of cropping–tillage practices on soil microbial community varies significantly between control and fertilized soils. For instance, under fertilization, each cropping–tillage practice exhibited unique dominant bacterial phyla with relatively large differences among them. In contrast, in control soils, the bacterial community compositions across cropping–tillage practices were more similar, showing smaller differences (Figure S6). Fungal differences among cropping–tillage practices in fertilized soils were primarily reflected in Mortierellomycota, whereas in control soils, differences were mainly associated with Basidiomycota (Figure S6). Similar patterns emerged in indicator species composition. Specifically, in fertilized soils, a greater number of bacterial indicator species associated with cropping–tillage practices were identified compared to control soils (Figure S9A,B; Table S6). Conversely, for fungal indicator species, fewer were identified in fertilized soils, whereas more were found in control soils (Figure S9C,D; Table S7). These results indicate that in the agricultural practices of this study, fertilization significantly alters the composition and structure of the soil microbial community through its pronounced effects on soil properties [86,87]. Consequently, fertilization interfered with the soil microbial community-shaping effects of cropping–tillage practices, resulting in significant differences between control and fertilized soils.

### 4.3. Soil Bacterial and Fungal Indicator Species Are Closely Related to the Maize Yield

We identified and screened microbial taxa that were strongly and significantly associated with maize grain yields, and their relative abundance showed significant positive or negative correlations with yields. Among these correlated microbial taxa, we further selected taxa belonging to indicator species.

Under both control and fertilized treatments, despite substantial differences in composition and potential ecological functions among these cropping–tillage-associated indicator species, all maintained significant correlations with maize grain yields (Figure 6). Based on the significant positive or negative correlations, we grouped these bacterial and fungal indicator species under different fertilizer treatments (Tables S8 and S9). These indicator species in distinct groups demonstrated potential as bioindicators of productivity for different cropping–tillage practices. Monitoring these indicator species via sequencing or functional gene amplification provides early warnings of impending nutrient limitations before significant crop impact, offering a scientific basis for precise and sustainable nutrient management. For example, specific species that are closely associated with soil nitrogen cycling processes serve as potential early bioindicators of soil nitrogen availability [88]. Likewise, the abundance of phosphorus-solubilizing microorganisms or phosphorus metabolic functional genes serves as a potential indicator of soil phosphorus availability [89]. Furthermore, microbial community parameters, particularly the relative abundance of specific phyla or functional genes, can provide integrated insights into soil nutritional status [86].

A total of 21 and 24 bacterial indicator species were identified as dominant in control and fertilized soils, respectively (Figure S9A,B; Table S6). These species predominantly participate in soil organic matter degradation, nutrient cycling, and ecological balance maintenance. For example, *Prevotellaceae* and *Escherichia* rapidly decompose and utilize labile organic matter [90,91]; *Microvirga* participates in nitrogen fixation [92]; and *Lactobacillus* promotes mineral dissolution through acid production, thereby indirectly enhancing plant nutrient uptake efficiency [93]. Specifically, in control soils, the primary correlated bacterial indicator species included *Dongia*, *Bacteroides*, *Prevotella_7*, *Sutterella*, *Ruminiclostridium_6*, *Lysobacter*, *Gemmatirosa*, and *Candidatus_Alysiosphaera* at the genus level. In fertilized soils, indicator species comprised *Kribbella*, *Terrimonas*, *Rhodomicrobium*, *Dongia*, *Dinghuibacter*, *Haliangium*, *Nocardioides*, *MND1*, *Nostoc_PCC*, and *Gemmatimonas*. The fungal indicator species related to grain yields included *Fusicolla* and an unidentified *Sordariomycete* in control soils, with *Pulvinula* and *Mortierella* in fertilized soils. Among these bacterial indicator species, *Dongia* fixes nitrogen, converting it into available ammonium nitrogen for plants. In the nitrogen cycle [94], *Nocardioides* converts organic nitrogen into inorganic nitrogen, improving soil nitrogen availability [95]. *Gemmatirosa* participates in phosphorus activation and storage, mineralizing organic phosphorus or dissolving insoluble phosphates [96]. *Terrimonas* decomposes and utilizes organic matter, converting it into plant-available nutrients [97]. *Rhodomicrobium* enhances nutrient availability for crop growth in the rhizosphere, improving soil microecology [98]. *Ruminiclostridium_6* produces butyric acid as an energy source for plant roots, promoting root development [99]. Siderophores produced by *Lysobacter* enhance drought and disease resistance [100]. *Haliangium* interacts with rhizosphere microorganisms [101], while *Kribbella* inhibits some plant pathogens, providing crop protection [102]. These indicator species participate in nutrient cycling, regulate crop growth, and protect crops against stress, directly or indirectly affecting maize grain yields. Results indicate that in multiple agricultural practice strategies, different tillage practices can differentially impact soil microorganisms [55,103]. Tillage practices alter the relationship between indicator species and crop productivity by modifying the soil microbial community. Therefore, potential interference among different agricultural practices must be carefully considered when selecting bioindicators for productivity evaluation.

## 5. Conclusions

Accurate evaluation of soil productivity in the field benefits the selection of appropriate agricultural practices based on production needs, which can reduce resource waste and environmental pollution from excessive inputs. Our findings clarify that microbial communities serve as sensitive bioindicators, mirroring the impact of agricultural practices on soil disturbance and maize grain yields through specific taxonomic groups. Furthermore, fertilization reconfigures the indicator species mediated by cropping–tillage practices, which show significant associations with maize productivity. However, the applicability of these indicator species across other cropping systems (e.g., wheat), soil types (e.g., black soil), and moisture conditions (e.g., variable irrigation regimes) requires empirical validation and further functional exploration by integrating metagenomic and metabolomic data. Therefore, appropriate indicator species or species combinations should be selected according to variations in agricultural practices. Moreover, integrating soil properties with bioindicators for soil fertility assessments would enable a more comprehensive evaluation of agricultural practices.

## Figures and Tables

**Table 1:** Variations in soil properties across different cropping–tillage practices under control and fertilized treatments based on linear mixed-effects models. Statistical significance of these cropping–tillage practice differences was assessed using type II ANOVA with Kenward–Roger approximation of the degrees of freedom. Bold values indicate significant variations.

Soil Properties	Fertilizer	Cropping–Tillage Practice				R^2^m	R^2^c
		CTMC	CTIC	RFMC	RFIC		
Moisture (%)	Control	16.89 ± 0.83 a	17.17 ± 0.12 a	17.84 ± 0.47 a	17.35 ± 0.26 a	0.114	0.307
	Fertilized	16.52 ± 0.22 a	17.30 ± 0.51 a	17.45 ± 0.28 a	17.56 ± 0.46 a	0.226	0.410
pH	Control	8.41 ± 0.03 a	8.39 ± 0.03 a	8.43 ± 0.01 a	8.39 ± 0.03 a	0.095	0.646
	Fertilized	8.37 ± 0.02 a	8.32 ± 0.06 a	8.34 ± 0.02 a	8.35 ± 0.02 a	0.075	0.506
TC (g/kg)	Control	17.28 ± 0.30 a	17.20 ± 0.32 a	17.03 ± 0.37 a	16.91 ± 0.43 a	0.039	0.384
	Fertilized	17.09 ± 0.21 a	17.19 ± 0.31 a	16.88 ± 0.09 a	16.94 ± 0.13 a	0.091	0.519
TN (g/kg)	Control	0.95 ± 0.02 a	0.95 ± 0.03 a	0.93 ± 0.02 a	0.95 ± 0.03 a	0.045	0.733
	Fertilized	0.96 ± 0.02 a	0.99 ± 0.02 a	0.99 ± 0.01 a	0.94 ± 0.02 a	0.258	0.258
C:N	Control	18.13 ± 0.16 a	18.22 ± 0.28 a	18.46 ± 0.20 a	17.80 ± 0.28 a	0.212	0.551
	Fertilized	17.76 ± 0.12 a	17.43 ± 0.29 a	17.14 ± 0.24 a	18.00 ± 0.27 a	0.033	0.033
DOC (mg/kg)	Control	65.92 ± 14.27 a	72.96 ± 17.08 a	64.52 ± 13.89 a	75.85 ± 15.04 a	0.025	0.250
	Fertilized	36.86 ± 15.01 a	28.66 ± 9.63 a	24.13 ± 8.39 a	30.68 ± 8.49 a	0.046	0.046
NH_4_^+^-N (mg/kg)	Control	**2.22 ± 0.14** b	**1.61 ± 0.09** a	**2.29 ± 0.20** b	**2.26 ± 0.14** b	0.490	0.807
	Fertilized	2.38 ± 0.38 a	2.98 ± 0.18 a	3.28 ± 0.31 a	3.31 ± 0.33 a	0.279	0.370
NO_3_^−^-N (mg/kg)	Control	1.75 ± 0.18 a	1.52 ± 0.08 a	1.73 ± 0.30 a	1.62 ± 0.16 a	0.060	0.461
	Fertilized	2.39 ± 0.42 a	2.36 ± 0.28 a	2.05 ± 0.24 a	1.94 ± 0.34 a	0.085	0.085
MBC (mg/kg)	Control	132.74 ± 16.69 a	140.55 ± 13.31 a	131.75 ± 6.51 a	133.28 ± 21.71 a	0.013	0.605
	Fertilized	162.50 ± 16.96 a	178.97 ± 17.66 a	170.30 ± 4.25 a	175.13 ± 12.58 a	0.049	0.334
MBN (mg/kg)	Control	28.30 ± 4.29 a	26.80 ± 2.83 a	24.99 ± 4.02 a	27.58 ± 2.32 a	0.033	0.461
	Fertilized	32.43 ± 5.02 a	30.15 ± 1.89 a	33.51 ± 2.76 a	33.84 ± 2.40 a	0.050	0.113

All values are shown as mean ± standard error. The linear mixed-effects model formula was soil properties ~ cropping–tillage practice + (1|block). R^2^m (marginal R^2^) represents the variance explained by fixed effects only; R^2^c (conditional R^2^) represents the variance explained by both fixed and random effects. Different lowercase letters within rows indicate significant differences between cropping–tillage practices. TC/TN: total carbon/nitrogen; C:N: carbon and nitrogen ratio; DOC: dissolved organic carbon; NH_4_^+^-N/NO_3_^−^-N: ammonium/nitrate-nitrogen; MBC/MBN: microbial biomass carbon/nitrogen.

**Table 2:** Variations in ASV richness and Shannon index of bacterial and fungal alpha diversity across different cropping–tillage practices under control and fertilized treatments based on linear mixed-effects models. Statistical significance of these cropping–tillage practice differences was assessed using type II ANOVA with Kenward–Roger approximation of the degrees of freedom. Bold values indicate significant variations.

Alpha Diversity		Fertilizer	Cropping–Tillage Practices				R^2^m	R^2^c
			CTMC	CTIC	RFMC	RFIC		
Bacteria	Richness	Control	1069.75 ± 41.41 a	1210.75 ± 54.07 a	1055.25 ± 71.48 a	1228.00 ± 46.68 a	0.357	0.357
		Fertilized	1214.75 ± 54.76 a	1211.50 ± 60.29 a	1129.75 ± 27.41 a	1300.00 ± 32.95 a	0.313	0.313
	Shannon	Control	6.29 ± 0.25 a	6.67 ± 0.04 a	6.34 ± 0.17 a	6.53 ± 0.09 a	0.202	0.295
		Fertilized	**6.67 ± 0.05** b	**6.63 ± 0.06** ab	**6.37 ± 0.10** a	**6.74 ± 0.02** b	0.563	0.617
Fungi	Richness	Control	391.25 ± 14.64 a	359.00 ± 8.75 a	373.75 ± 19.44 a	363.25 ± 3.20 a	0.196	0.511
		Fertilized	**315.00 ± 8.98** a	**314.25 ± 17.31** a	**365.00 ± 15.70** b	**328.00 ± 15.72** ab	0.342	0.694
	Shannon	Control	3.90 ± 0.13 a	4.09 ± 0.11 a	4.21 ± 0.06 a	4.03 ± 0.02 a	0.294	0.294
		Fertilized	**3.85 ± 0.11** bc	**3.72 ± 0.16** ab	**3.96 ± 0.12** c	**3.57 ± 0.16** a	0.233	0.881

All values are shown as mean ± standard error. The linear mixed-effects model formula was alpha diversity ~ cropping–tillage practice + (1|block). R^2^m (marginal R^2^) represents the variance explained by fixed effects only; R^2^c (conditional R^2^) represents the variance explained by both fixed and random effects. Different lowercase letters within rows indicate significant differences between cropping–tillage practices.

## Data Availability

The original contributions presented in this study are included in the article/Supplementary Material. Further inquiries can be directed to the corresponding authors.

## Supplementary Materials

The following supporting information can be downloaded at: https://www.mdpi.com/article/10.3390/microorganisms13061384/s1, Figure S1: Meteorological observation parameters of the experimental field; Figure S2: Variations in soil properties between control and fertilized treatments; Figure S3: Variations in plant height, straw weight, and grain weight between control and fertilized treatments; Figure S4: Rarefaction curves of bacterial and fungal communities for each sample; Figure S5: Taxonomic profiles of bacterial and fungal communities at the phylum level for each sample; Figure S6: Variations in dominant bacterial phyla across different cropping–tillage practices in control and fertilized soils; Figure S7: Variations in dominant fungal phyla across different cropping–tillage practices in control and fertilized soils; Figure S8: Shared and exclusive bacterial and fungal dominant taxa in control and fertilized soils; Figure S9: Dominant and sensitive profiles of bacterial and fungal indicator species in control and fertilized soils; Figure S10: Shared and exclusive bacterial and fungal sensitive indicator species between control and fertilized soils; Figure S11: Distance–decay curve for bacterial and fungal Bray–Curtis dissimilarity against variation in maize productivity between each sample; Table S1: Soil properties before field experiment setup; Table S2: Effects of fertilizer, cropping–tillage practice, and their interactions on soil properties; Table S3: Effects of fertilizer, cropping–tillage practice, and their interactions on maize productivity; Table S4: Effects of fertilizer, cropping–tillage practices, and their interactions on Bray–Curtis dissimilarities of bacterial and fungal communities via PERMANOVA and ANOSIM; Table S5: PERMANOVA and ANOSIM analyses of bacterial and fungal communities between different cropping–tillage practices in control and fertilized soils; Table S6: Dominant bacterial indicator species in control and fertilized soils; Table S7: Dominant fungal indicator species in control and fertilized soils; Table S8: Significant correlations between the relative abundance of bacterial indicator species and maize grain yields calculated by Spearman’s correlation coefficient; Table S9: Significant correlations between the relative abundance of fungal indicator species and maize grain yields calculated by Spearman’s correlation coefficient.
