# Supplementary material for: Fertilization Alters Indicator Species Serving as Bioindicators for Evaluating Agricultural Practices Related to Maize Grain Yield
Source: Microorganisms. 2025 Jun 13;13(6):1384. doi: 10.3390/microorganisms13061384 (PMC12195582; doi:10.3390/microorganisms13061384)
Supplement: Supplementary file 1 [file microorganisms-13-01384-s001.zip › microorganisms-3625610-supplementary.pdf]

Supplementary material for

Fertilization Alters Indicator Species Serving as Bioindicators for Evaluating Agricultural Practices Related to Maize Grain Yield

Guoqiang Li, Jiaqing Liu, Wenya Zhang, Jvshui Hu, Peng Shi *, Gehong Wei *

State Key Laboratory for Crop Stress Resistance and High-Efficiency Production, Shaanxi Key Laboratory of Agricultural and Environmental Microbiology, College of Life Sciences, Northwest A&F University, Yangling 712100, China; guoqiangli@nwafu.edu.cn (G.L.); 18740411203@163.com (J.L.); 2023056378@nwafu.edu.cn (W.Z.); hujvshui@163.com (J.H.)

* Correspondence: shipeng27@nwafu.edu.cn (P.S.); weigehong@nwsuaf.edu.cn (G.W.); Tel.: +86-135-7227-7787 (P.S.)

Table S1. Soil properties before field experiment setup. Differences between blocks were estimated by Kruskal-Wallis tests. Significant effects are indicated in bold at $p < 0.05$.

Block	pH	OM (g/kg)	TN (g/kg)	TP (g/kg)	AN (mg/kg)	AP (mg/kg)
Block I	8.19±0.04	15.86±0.65	1.06±0.09	1.58±0.02	48.54±1.57	23.24±2.26
Block II	8.28±0.02	16.33±0.79	0.90±0.05	1.64±0.08	50.48±1.71	25.54±2.83
Block III	8.26±0.01	17.16±0.82	0.98±0.01	1.66±0.05	55.05±3.94	26.56±2.29
Block IV	8.25±0.02	17.38±0.43	0.95±0.05	1.88±0.08	49.42±2.57	30.06±3.12
P-value	0.216	0.385	0.393	0.052	0.528	0.441

All data are shown as mean ± standard error. OM: organic matter; TN: total nitrogen; TP: total phosphorus; AN: available nitrogen; AP: available phosphorus; AK: available potassium.

Table S2. Effects of fertilizer, cropping-tillage practice, and their interactions on soil properties based on linear mixed-effects models. Statistical significance was assessed using type II ANOVA with Kenward-Rodger approximation of the degrees of freedom. Significant effects are indicated in bold.

Soil properties	Fertilizer		Cropping-tillage		Fertilizer:Cropping		R^2m	R^2c
	F value	Pr (> *F*)	*F* value	Pr (> *F*)	*F* value	Pr (> *F*)		
Moisture (%)	0.122	0.731	1.882	0.164	0.285	0.835	0.158	0.259
pH	15.031	**<0.001**	0.894	0.461	0.818	0.499	0.254	0.609
TC (g/kg)	0.301	0.589	0.977	0.422	0.135	0.938	0.055	0.530
TN (g/kg)	3.917	0.061	0.456	0.716	1.618	0.215	0.176	0.462
TC/TN	11.454	**0.003**	0.159	0.923	3.686	**0.028**	0.426	0.426
DOC (mg/kg)	18.770	**<0.001**	0.180	0.909	0.163	0.920	0.385	0.398
NH_4^+-N (mg/kg)	36.117	**<0.001**	3.619	**0.030**	3.011	0.053	0.574	0.682
NO_3^--N (mg/kg)	7.650	**0.012**	0.400	0.754	0.451	0.719	0.248	0.248
MBC (mg/kg)	22.020	**<0.001**	0.425	0.737	0.107	0.955	0.304	0.600
MBN (mg/kg)	5.504	**0.029**	0.188	0.904	0.241	0.867	0.180	0.180

The linear mixed model formula was: soil properties ~ fertilizer + cropping-tillage practices + fertilizer:cropping-tillage practices + (1|block). R^2m (Marginal R^2) represents the variance explained by fixed effects only; R^2c (Conditional R^2) represents the variance explained by both fixed and random effects. TC/TN: total carbon/nitrogen; C:N: carbon and nitrogen ratio; DOC: dissolved organic carbon; NH_4^+-N/NO_3^--N: ammonium/nitrate-nitrogen; MBC/MBN: microbial biomass carbon/nitrogen.

Table S3. Effects of fertilizer, cropping-tillage practice, and their interactions on maize productivity based on linear mixed-effects models. Statistical significance was assessed using type II ANOVA with Kenward-Rodger approximation of the degrees of freedom. Significant effects are indicated in bold.

Maize productivity	Fertilizer		Cropping-tillage		Fertilizer : Cropping		R^2m	R^2c
	F value	Pr (> *F*)	*F* value	Pr (> *F*)	*F* value	Pr (> *F*)		
Plant height (cm)	175.69	**< 0.001**	37.07	**< 0.001**	2.29	0.081	0.649	0.649
Straw weight (g plant^{-1})	53.18	**< 0.001**	13.30	**< 0.001**	0.36	0.782	0.367	0.381
Grain weight (g plant^{-1})	85.67	**< 0.001**	6.93	**< 0.001**	0.95	0.421	0.634	0.634

The linear mixed model formula was: maize productivity ~ fertilizer + cropping-tillage practice + fertilizer : cropping-tillage practice + (1|block). R^2m (Marginal R^2) represents the variance explained by fixed effects only; R^2c (Conditional R^2) represents the variance explained by both fixed and random effects.

Table S4. Effects of fertilizer, cropping–tillage practices, and their interactions on Bray-Curtis dissimilarities of bacterial and fungal communities via PERMANOVA and ANOSIM. Significant effects are indicated in bold.

PERMANOVA	Bacteria		Fungi	
	R^2	*P*	R^2	*P*
Fertilizer	0.081	**< 0.001**	0.064	**0.002**
Cropping–tillage practices	0.114	**0.032**	0.142	**0.002**
Fertilizer : Cropping	0.162	**< 0.001**	0.132	**0.005**
**ANOSIM**	**Bacteria**		**Fungi**	
	R	*P*	R	*P*
Fertilizer	0.168	**0.002**	0.112	**0.007**
Cropping–tillage practices	0.119	**0.008**	0.120	**0.009**

Table S5. PERMANOVA and ANOSIM analyses of bacterial and fungal communities between different cropping–tillage practices in control and fertilized soils, respectively. Significant effects are indicated in bold.

	Fertilizer	PERMANOVA		ANOSIM	
		R^2	*P*	R	*P*
Bacteria	Control	0.252	**0.038**	0.203	**0.012**
	Fertilized	0.356	**<0.001**	0.388	**0.001**
Fungi	Control	0.303	**0.006**	0.291	**0.004**
	Fertilized	0.299	**0.004**	0.307	**0.003**

Table S6. Dominant bacterial indicator species in control and fertilized soils.

ASV_ID		Phylum	Class	Genus
Control	ASV_9	Fusobacteria	Fusobacteriia	*Fusobacterium*
	ASV_17	Acidobacteria	Blastocatellia	--
	ASV_22	Firmicutes	Bacilli	*Bacillus*
	ASV_23	Acidobacteria	Blastocatellia	--
	ASV_24	Acidobacteria	Subgroup_6	--
	ASV_42	Acidobacteria	Blastocatellia	*RB41*
	ASV_43	Proteobacteria	Alphaproteobacteria	*Microvirga*
	ASV_48	Proteobacteria	Alphaproteobacteria	*Sphingomonas*
	ASV_51	Firmicutes	Bacilli	*Bacillus*
	ASV_66	Chloroflexi	Chloroflexia	--
	ASV_85	Firmicutes	Bacilli	--
	ASV_115	Chloroflexi	Dehalococcoidia	--
	ASV_131	Proteobacteria	Gammaproteobacteria	*MND1*
	ASV_137	Acidobacteria	Subgroup_6	--
	ASV_141	Acidobacteria	Blastocatellia	--
	ASV_146	Acidobacteria	Subgroup_6	--
	ASV_151	Chloroflexi	TK10	--
	ASV_159	Firmicutes	Bacilli	*Bacillus*
	ASV_201	Proteobacteria	Alphaproteobacteria	*Rhizobium*
	ASV_207	Bacteroidetes	Bacteroidia	*Chryseolinea*
	ASV_363	Actinobacteria	Actinobacteria	*Streptomyces*
Fertilized	ASV_2	Firmicutes	Clostridia	*Romboutsia*
	ASV_3	Proteobacteria	Gammaproteobacteria	*Escherichia/Shigella*
	ASV_6	Firmicutes	Clostridia	*Terrisporobacter*
	ASV_9	Fusobacteria	Fusobacteriia	*Fusobacterium*
	ASV_15	Proteobacteria	Gammaproteobacteria	*MND1*
	ASV_18	Proteobacteria	Alphaproteobacteria	*Sphingomonas*
	ASV_35	Proteobacteria	Alphaproteobacteria	*Nordella*
	ASV_43	Proteobacteria	Alphaproteobacteria	*Microvirga*
	ASV_58	Proteobacteria	Deltaproteobacteria	--
	ASV_72	Bacteroidetes	Bacteroidia	*dgA-11*
	ASV_75	Actinobacteria	Acidimicrobiia	--
	ASV_76	Acidobacteria	Subgroup_6	--
	ASV_78	Bacteroidetes	Bacteroidia	*Prevotellaceae_UCG-003*
	ASV_95	Fusobacteria	Fusobacteriia	*Fusobacterium*
	ASV_96	Gemmatimonadetes	Gemmatimonadetes	--
	ASV_116	Firmicutes	Clostridia	*Clostridium*
	ASV_145	Actinobacteria	Thermoleophilia	--
	ASV_152	Proteobacteria	Alphaproteobacteria	--
	ASV_164	Firmicutes	Bacilli	*Lactobacillus*
	ASV_181	Gemmatimonadetes	Gemmatimonadetes	--
	ASV_189	Chloroflexi	Gitt-GS-136	--
	ASV_192	Chloroflexi	Chloroflexia	--
	ASV_221	Bacteroidetes	Bacteroidia	*Rikenellaceae_RC9*
	ASV_242	Bacteroidetes	Bacteroidia	--

Note: "--" indicated that the species was not identified.

Table S7. Dominant fungal indicator species in control and fertilized soils.

ASV_ID		Phylum	Class	Genus
**Control**	ASV_3	Basidiomycota	Tremellomycetes	*Cryptococcus*
	ASV_2	Ascomycota	Sordariomycetes	*Gibberella*
	ASV_18	--	--	--
	ASV_25	Ascomycota	Sordariomycetes	*Humicola*
	ASV_49	Ascomycota	--	--
	ASV_81	Basidiomycota	Tremellomycetes	*Cryptococcus*
	ASV_84	Mortierellomycota	Mortierellomycetes	*Mortierella*
	ASV_131	Ascomycota	Ascomycota	*Tetracladium*
**Fertilized**	ASV_2	Ascomycota	Sordariomycetes	*Gibberella*
	ASV_1	Ascomycota	Sordariomycetes	--
	ASV_10	Ascomycota	Dothideomycetes	*Cladosporium*
	ASV_12	Ascomycota	Dothideomycetes	--
	ASV_15	Mortierellomycota	Mortierellomycetes	*Mortierella*

Note: "--" indicated that the species was not identified.

Table S8. Significant correlations between the relative abundance of bacterial indicator species and maize grain yields calculated by Spearman's correlation coefficient

**ASVID**		***r***	***P***	**Phylum**	**Family**	**Genus**
**Positive** in control soils (Group 1)	ASV_1117	0.59	0.016	Proteobacteria	BIrii41	--
	ASV_1258	0.59	0.017	Proteobacteria	Dongiaceae	*Dongia*
	ASV_1319	0.66	0.006	Bacteroidetes	Muribaculaceae	--
	ASV_1369	0.65	0.006	Firmicutes	Lachnospiraceae	*Lachnospiraceae_NK4A136*
	ASV_1375	0.58	0.019	Bacteroidetes	Bacteroidaceae	*Bacteroides*
	ASV_1413	0.56	0.024	Bacteroidetes	Prevotellaceae	*Prevotella_7*
	ASV_1487	0.54	0.032	Proteobacteria	Burkholderiaceae	*Sutterella*
	ASV_1601	0.62	0.011	Firmicutes	Ruminococcaceae	*Ruminiclostridium_6*
	ASV_1602	0.63	0.009	Firmicutes	Ruminococcaceae	--
	ASV_1788	0.51	0.043	Firmicutes	Erysipelotrichaceae	*Erysipelotrichaceae_UCG-004*
	ASV_1879	0.73	0.001	Firmicutes	Clostridiales_vadinBB60	--
	ASV_1887	0.75	0.001	Bacteroidetes	Muribaculaceae	--
	ASV_1895	0.77	0.001	Firmicutes	Ruminococcaceae	*Ruminococcus_1*
	ASV_1906	0.75	0.001	Bacteroidetes	Muribaculaceae	--
	ASV_2246	0.65	0.006	Actinobacteria	Eggerthellaceae	--
	ASV_2307	0.55	0.027	Chloroflexi	--	--
	ASV_2335	0.57	0.021	Proteobacteria	Xanthomonadaceae	*Lysobacter*
	ASV_2450	0.63	0.009	Firmicutes	Staphylococcaceae	*Staphylococcus*
	ASV_2486	0.62	0.010	Gemmatimonadetes	Gemmatimonadaceae	*Gemmatirosa*
	ASV_2673	0.68	0.004	Proteobacteria	Methylophilaceae	*MM2*
	ASV_426	0.57	0.022	Firmicutes	Aerococcaceae	*Aerococcus*
	ASV_609	0.67	0.005	Actinobacteria	Iamiaceae	*Iamia*
	ASV_748	0.54	0.032	Firmicutes	Erysipelotrichaceae	*Allobaculum*
	ASV_762	0.62	0.010	Acidobacteria	--	--
	ASV_824	0.62	0.011	Gemmatimonadetes	Gemmatimonadaceae	--
	ASV_905	0.76	0.001	Bacteroidetes	Bacteroidaceae	*Bacteroides*
	ASV_972	0.65	0.006	Bacteroidetes	Bacteroidaceae	*Bacteroides*
**Negative** in control soils (Group 2)	ASV_1783	-0.52	0.041	Gemmatimonadetes	--	--
	ASV_1094	-0.53	0.034	Chloroflexi	--	--
	ASV_939	-0.59	0.017	Proteobacteria	--	--
	ASV_399	-0.59	0.016	Actinobacteria	Ilumatobacteraceae	--
	ASV_924	-0.64	0.007	Acidobacteria	--	--
	ASV_1958	-0.67	0.004	Proteobacteria	Geminicoccaceae	*Candidatus_Alysiosphaera*
**Positive** in fertilized soils (Group 3)	ASV_1077	0.82	0.000	Actinobacteria	Nocardioidaceae	*Kribbella*
	ASV_734	0.79	0.000	Proteobacteria	Rhizobiaceae	--
	ASV_825	0.77	0.000	Proteobacteria	Devosiaceae	--
	ASV_2383	0.73	0.001	Proteobacteria	--	--
	ASV_1388	0.70	0.003	Actinobacteria	--	--
	ASV_860	0.68	0.004	Proteobacteria	Syntrophaceae	--
	ASV_2918	0.68	0.004	Bacteroidetes	Chitinophagaceae	*Terrimonas*
	ASV_1489	0.67	0.005	Proteobacteria	Rhodomicrobiaceae	*Rhodomicrobium*
	ASV_1620	0.66	0.006	Bacteroidetes	AKYH767	--
	ASV_1311	0.64	0.007	Acidobacteria	--	--
	ASV_1684	0.62	0.010	Proteobacteria	Nitrosomonadaceae	*MND1*

	ASV_1216	0.62	0.011	Proteobacteria	Dongiaceae	*Dongia*
	ASV_1437	0.61	0.011	Chloroflexi	--	--
	ASV_1941	0.58	0.018	Proteobacteria	Amb-16S-1323	--
	ASV_1884	0.58	0.019	Acidobacteria	--	--
	ASV_2345	0.57	0.021	Bacteroidetes	Chitinophagaceae	*Dinghuibacter*
	ASV_249	0.55	0.027	Bacteroidetes	Saprospiraceae	--
	ASV_1573	0.51	0.044	Proteobacteria	Rhodomicrobiaceae	*Rhodomicrobium*
	ASV_2118	0.50	0.049	Proteobacteria	Haliangiaceae	*Haliangium*
**Negative** in fertilized soils (Group 4)	ASV_491	-0.52	0.041	Chloroflexi	JG30-KF-CM45	--
	ASV_1066	-0.53	0.034	Chloroflexi	--	--
	ASV_1590	-0.54	0.032	Actinobacteria	Nocardioidaceae	*Nocardioides*
	ASV_640	-0.54	0.029	Actinobacteria	Nocardioidaceae	*Nocardioides*
	ASV_1435	-0.56	0.024	Proteobacteria	Nitrosomonadaceae	*MND1*
	ASV_953	-0.57	0.023	Cyanobacteria	Nostocaceae	*Nostoc_PCC-73102*
	ASV_1208	-0.59	0.017	Cyanobacteria	Nostocaceae	*Nostoc_PCC-7107*
	ASV_2308	-0.59	0.017	Actinobacteria	Micromonosporaceae	--
	ASV_291	-0.61	0.013	Chloroflexi	JG30-KF-CM45	--
	ASV_1252	-0.65	0.006	Gemmatimonadetes	Gemmatimonadaceae	*Gemmatimonas*
	ASV_1264	-0.71	0.002	Chloroflexi	A4b	--

Note: "--" indicated that the species was not identified.

Table S9. Significant correlations between the relative abundance of fungal indicator species and maize grain yields calculated by Spearman's correlation coefficient

ASVID		*r*	*P*	Phylum	Family	Genus
**Positive** in control soils (Group 5)	ASV_96	0.74	0.001	--	--	--
	ASV_276	0.68	0.003	--	--	--
	ASV_239	0.67	0.005	Glomeromycota	Glomeraceae	*Glomus*
	ASV_474	0.60	0.015	--	--	--
	ASV_806	0.59	0.017	--	--	--
	ASV_189	0.54	0.031	Ascomycota	Nectriaceae	*Fusicolla*
**Negative** in control soils (Group 6)	ASV_792	-0.54	0.029	--	--	--
	ASV_940	-0.61	0.012	Ascomycota	--	--
**Positive** in fertilized soils (Group 7)	ASV_88	0.72	0.002	Ascomycota	--	--
	ASV_298	0.71	0.002	Ascomycota	--	--
	ASV_517	0.67	0.005	Ascomycota	Pyronemataceae	*Pulvinula*
	ASV_1	0.66	0.006	Ascomycota	Nectriaceae	--
	ASV_292	0.62	0.011	--	--	--
	ASV_1274	0.61	0.012	Ascomycota	Sympoventuriaceae	--
	ASV_98	0.55	0.028	Ascomycota	Stachybotryaceae	--
**Negative** in fertilized soils (Group 8)	ASV_15	-0.54	0.033	Mortierellomycota	Mortierellaceae	*Mortierella*

Note: "--" indicated that the species was not identified.

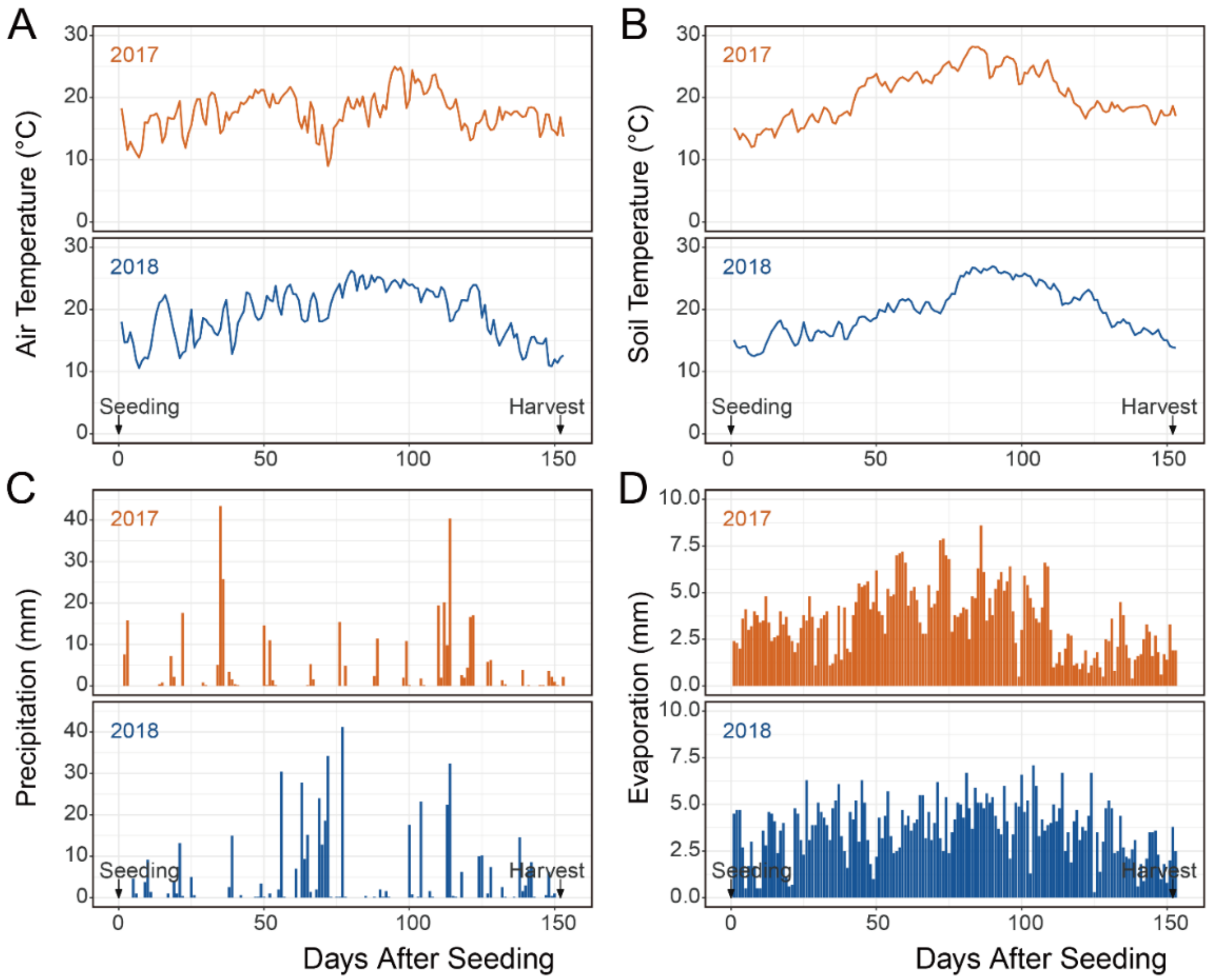

Figure S1. Meteorological observation parameters of the experimental filed. (A–D) Time course of daily air temperature (A), soil temperature (B), precipitation (C), and evaporation (D) during maize growing season in 2017 and 2018.

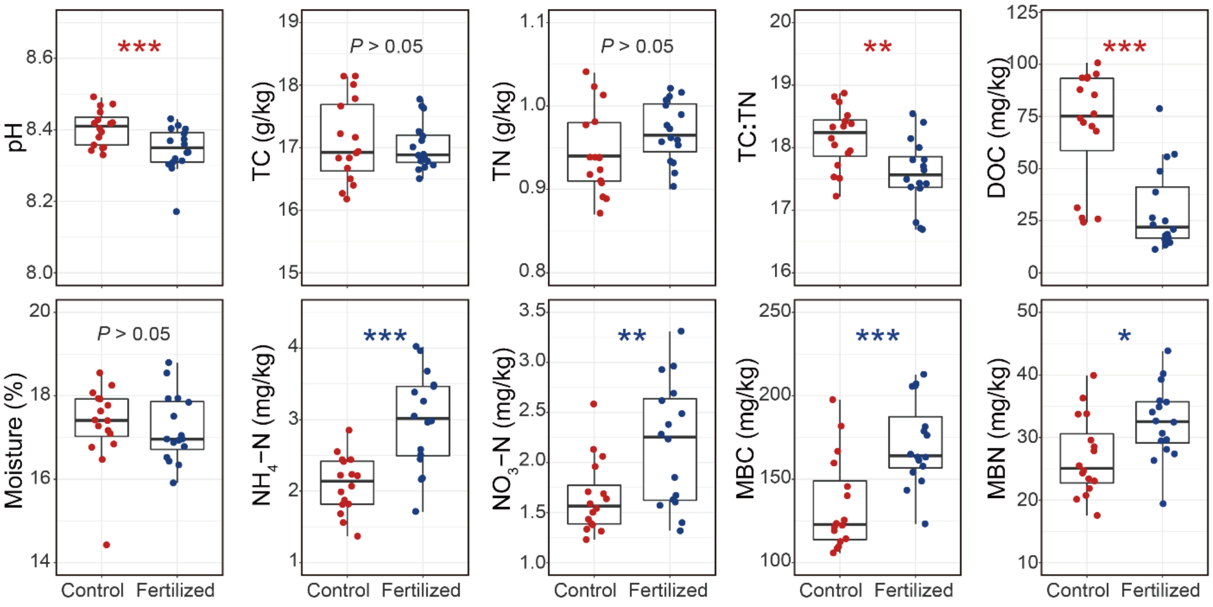

Figure S2. Variations in soil properties between control and fertilized treatments. The significance of differences was evaluated using Wilcoxon rank-sum test. Boxplots with asterisks indicate significant differences. ***, $P < 0.001$; **, $P < 0.01$; *, $P < 0.05$. TC/TN: total carbon/nitrogen; C:N: carbon and nitrogen ratio; DOC: dissolved organic carbon; NH_4^+-N/NO_3^--N: ammonium/nitrate-nitrogen; MBC/MBN: microbial biomass carbon/nitrogen.

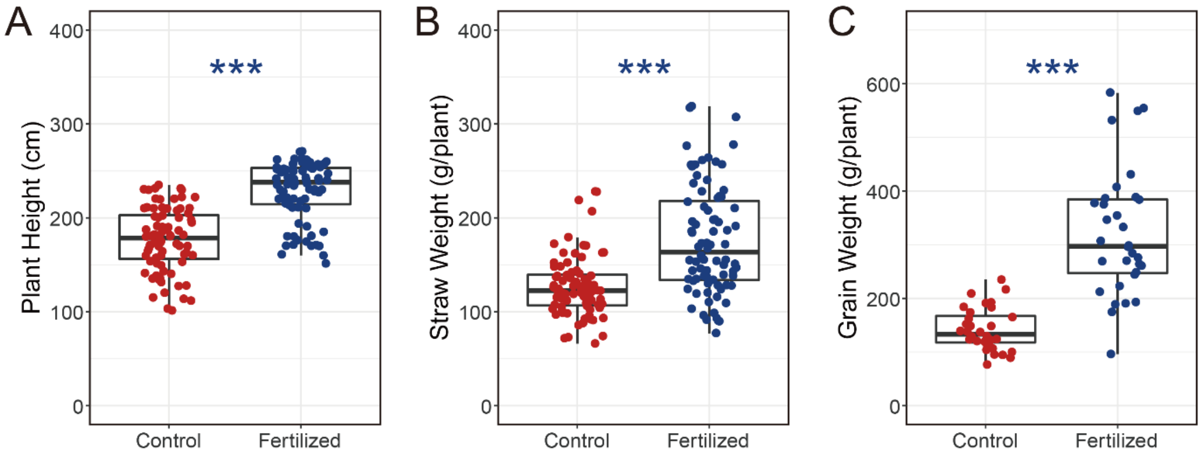

Figure S3. Variations in plant height (A), straw weight (B), and grain weight (C) between control and fertilized treatments. The significance of differences was evaluated using Wilcoxon rank-sum test. Boxplots with asterisks indicate significant differences. ***, $P < 0.001$.

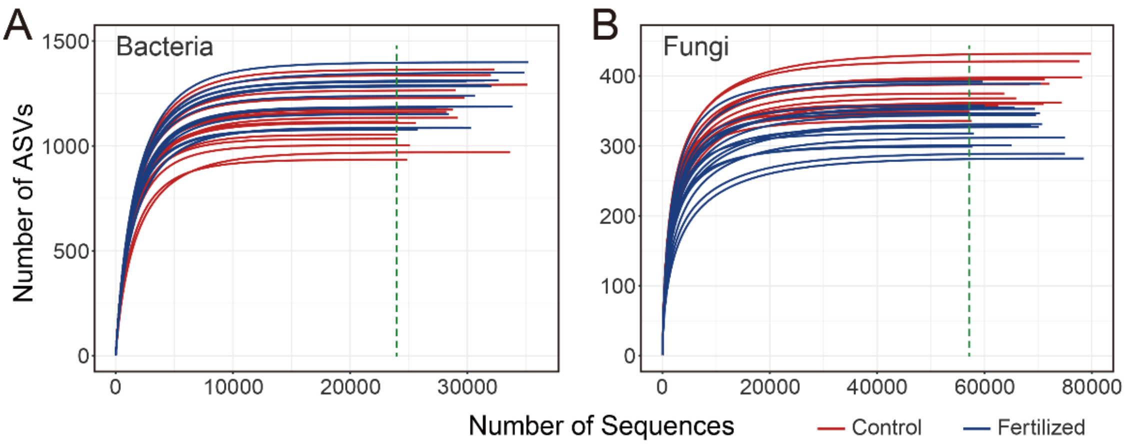

Figure S4. Rarefaction curves of bacterial (A) and fungal (B) communities for each sample. Red lines indicated control samples, and blue lines indicated fertilized samples. Both bacteria and fungi rarefaction curves reached the saturation stage with increasing sequencing depth, indicating that most microbes were captured. The green dashed lines indicated the rarefaction depth for each sample, rarefied to 23,675 and 57,219 counts per sample for bacterial and fungal communities, respectively.

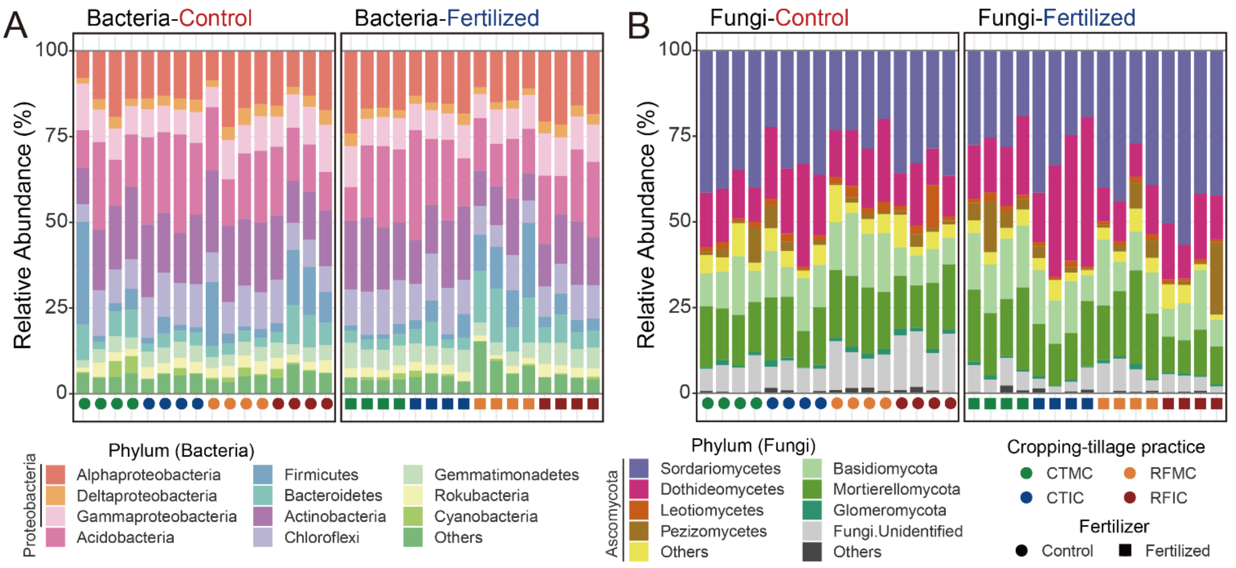

Figure S5. Taxonomic profiles of bacterial (A) and fungal (B) communities at the phylum level for each sample. Proteobacteria and Ascomycota were further split into their respective classes. Bacteria and fungi phyla with relative abundances less than 1% were summarized as “Others”. Cropping–tillage practice samples are indicated by colors, while fertilizer treatment samples are indicated by shapes.

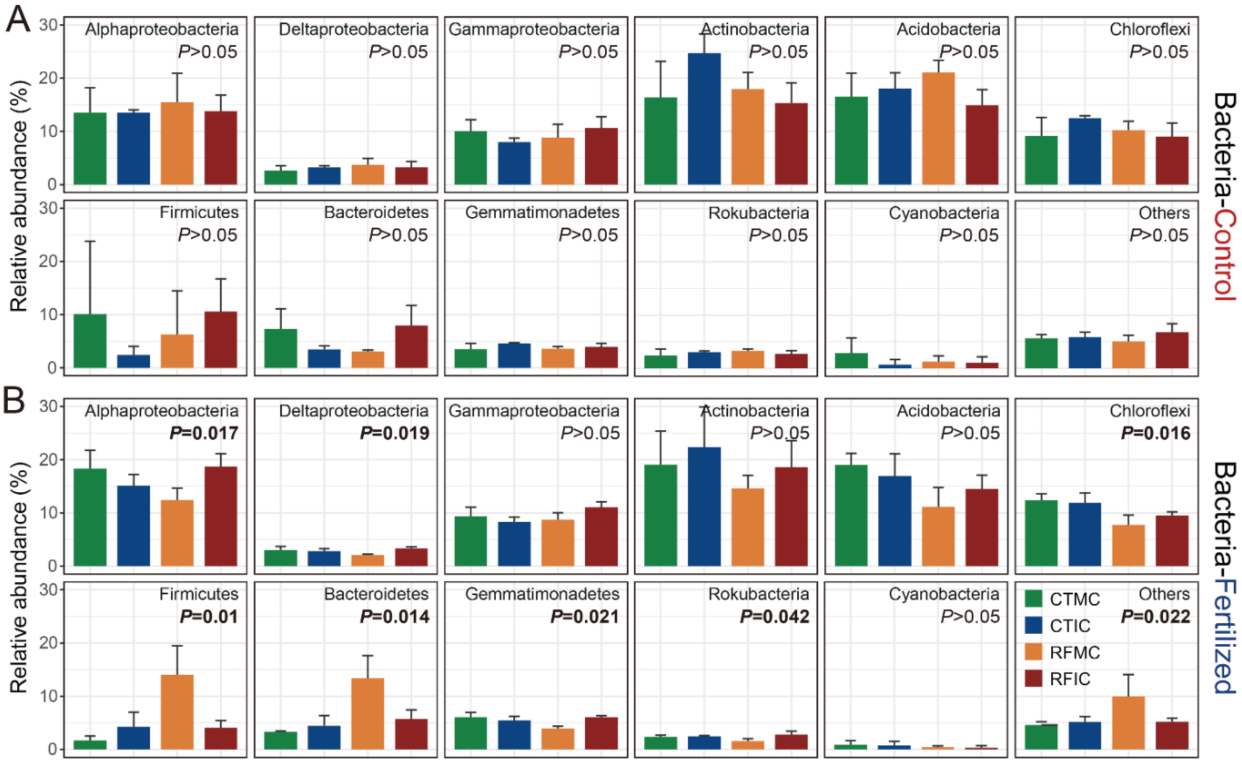

Figure S6. Variations in dominant bacterial phyla across different cropping-tillage practices in control (A) and fertilized (B) soils. Bar plots display the relative abundance of dominant phyla across cropping-tillage practices. Proteobacteria are subdivided into their respective classes. Phyla with relative abundances less than 1% are summarized as "Others". The effect across different cropping-tillage practices was estimated based on linear mixed-effects models. The linear mixed-effects model formula was: dominant phyla ~ cropping-tillage practice + (1 | block). Statistical significance was assessed using type II ANOVA with Kenward-Rodger approximation of the degrees of freedom. Significant effects are indicated in bold.

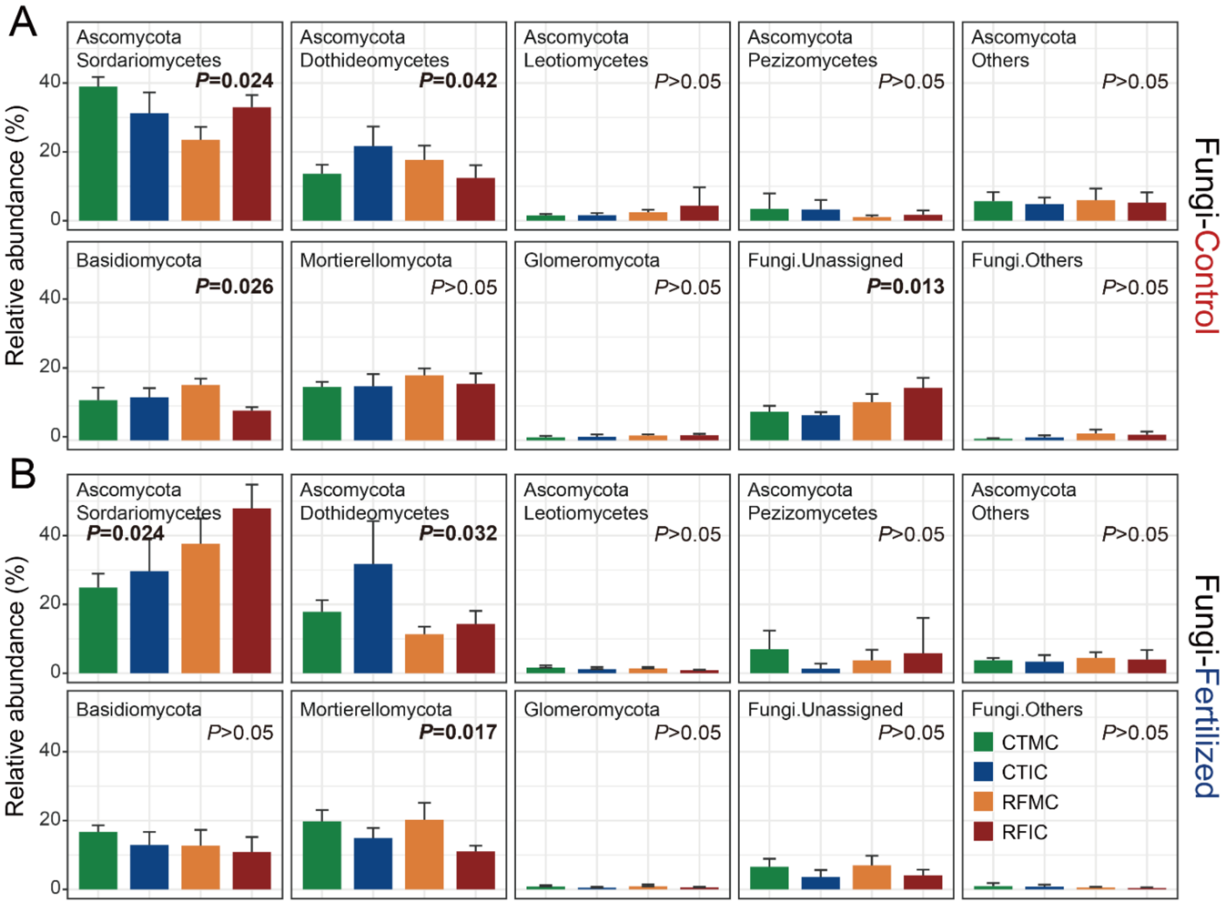

Figure S7. Variations in dominant fungal phyla across different cropping-tillage practices in control (A) and fertilized (B) soils. Bar plots display the relative abundance of dominant phyla across cropping-tillage practices. Ascomycota are split into their respective classes. Phyla with relative abundances less than 1% are summarized as "Fungi.Others". The effect across different cropping-tillage practices was estimated based on linear mixed-effects models. The linear mixed-effects model formula was: dominant phyla ~ cropping-tillage practice + (1|block). Statistical significance was assessed using type II ANOVA with Kenward-Rodger approximation of the degrees of freedom. Significant effects are indicated in bold.

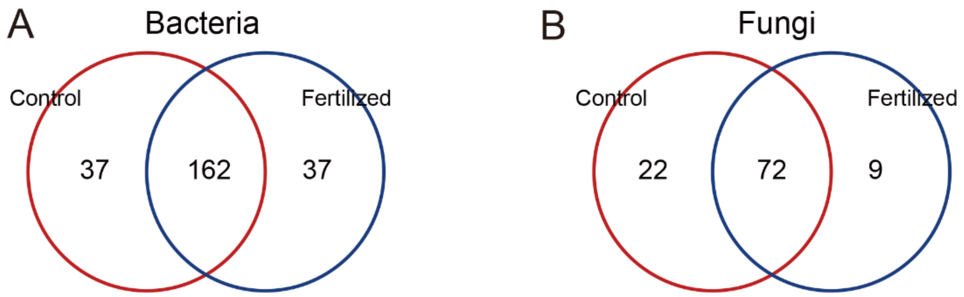

Figure S8. Shared and sole of bacterial (A) and fungal (B) dominant taxa in control and fertilized soils. Venn diagrams show the number of dominant taxa responding to cropping–tillage practices identified in control and fertilized soils.

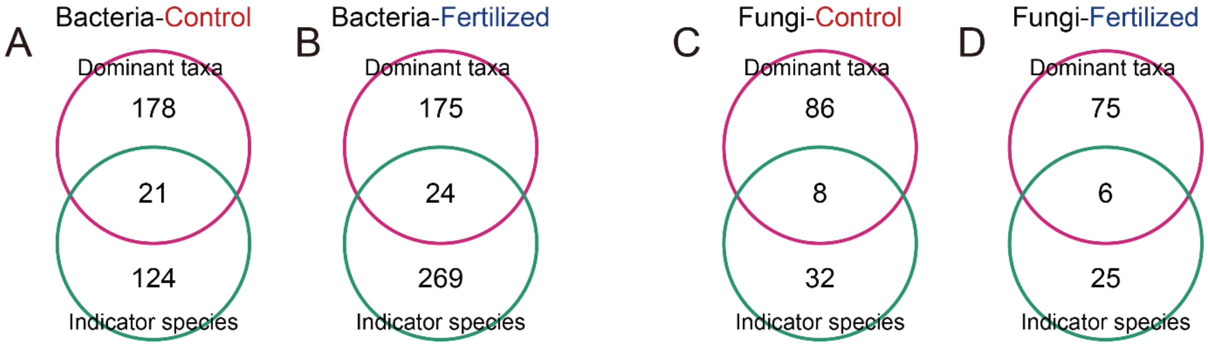

Figure S9. Dominant and sensitive profiles of bacterial (A–B) and fungal (C–D) indicator species in control and fertilized soils. Venn diagrams show the number of dominant and sensitive indicator species.

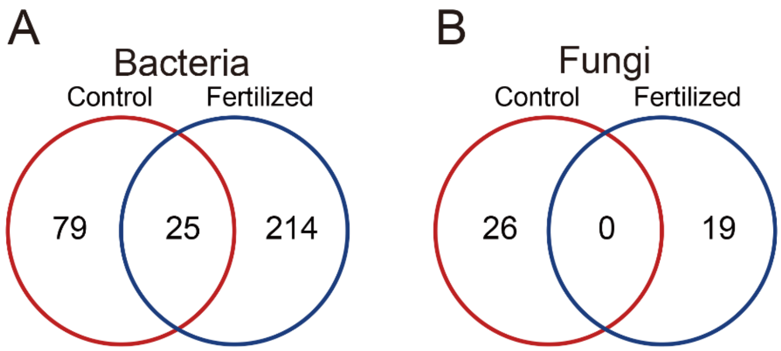

Figure S10. Shared and sole of bacterial (A) and fungal (B) sensitive indicator species between control and fertilized soils. Venn diagrams show the number of overlapping sensitive indicator species.

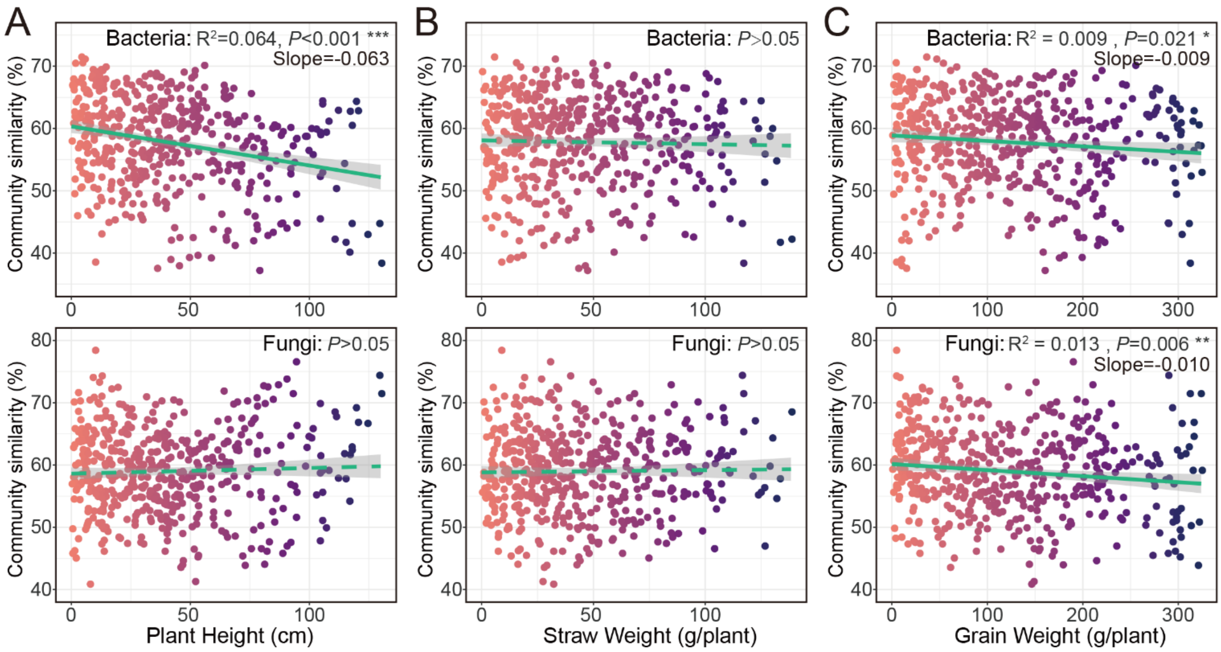

Figure S11. Distance–decay curve for bacterial and fungal Bray–Curtis dissimilarity against variation of maize productivity between each sample. Distance–decay curve for bacterial and fungal Bray–Curtis dissimilarity against variation in plant height (A), straw weight (B), and grain weight (C) are shown. Green lines denote the ordinary least squares linear regression. Asterisks represent significance of correlation. ***, $P < 0.001$; **, $P < 0.01$; *, $P < 0.05$.
